# Disruption of sphingolipid metabolism promotes tau seeding through endolysosomal membrane rigidification and rupture

**DOI:** 10.7554/eLife.106865

**Published:** 2026-08-27

**Authors:** Jessica Tittelmeier, Carl Alexander Sandhof, Nicole Martin, Deike El-Kabarity, Soki-Bradel Ngonza-Nito, Ronald Melki, Carmen Nussbaum-Krammer

**Affiliations:** 1 Chair of Neuroanatomy, Institute of Anatomy, Faculty of Medicine, Ludwig-Maximilians-University Munich (LMU Munich) Munich Germany; 2 Center for Molecular Biology of Heidelberg University (ZMBH) and German Cancer Research Center (DKFZ), DKFZ-ZMBH Alliance Heidelberg Germany; 3 Chemical Neurobiology Laboratory, Center for Genomic Medicine, Departments of Neurology and Psychiatry, Massachusetts General Hospital and Harvard Medical School Boston United States; 4 Institute Francois Jacob (MIRCen), CEA, and Laboratory of Neurodegenerative Diseases, CNRS Fontenay-Aux-Roses France; University of Groningen Netherlands; University of Cambridge United Kingdom

**Keywords:** Alzheimer's disease, tau propagation, sphingolipid metabolism, endolysosomal system, lipid homeostasis, membrane fluidity, *C. elegans*

## Abstract

Endolysosomal dysfunction is a hallmark of Alzheimer’s disease and related tauopathies, yet underlying mechanisms remain poorly understood. This study investigates the role of sphingolipid metabolism in maintaining endolysosomal membrane integrity and its impact on tau aggregation and toxicity in *Caenorhabditis elegans* and human cell culture models. Fluorescence recovery after photobleaching and C-Laurdan dye imaging revealed that silencing sphingolipid metabolism genes reduced endolysosomal vesicle membrane fluidity, increasing their rupture. The accumulation of aggregated tau in endolysosomal vesicles further aggravated endomembrane rigidification and damage, and promoted seeded tau aggregation, potentially by facilitating the escape of tau seeds from the endolysosomal system. Supplementation with unsaturated fatty acids improved membrane fluidity, suppressing endolysosomal rupture and seeded tau aggregation in cell models, and alleviating tau-associated neurotoxicity in *C. elegans*. Together, this study provides mechanistic insight into how perturbation of sphingolipid metabolism promotes endolysosomal membrane damage and contributes to the escape of aggregated tau from this compartment, suggesting that restoration of membrane fluidity may represent a strategy to limit tau propagation and toxicity.

## Introduction

The gradual accumulation of microtubule-associated protein tau (MAPT/tau) aggregates is a hallmark of Alzheimer’s disease (AD) and related tauopathies. Misfolded tau species exhibit prion-like behavior by self-replicating and spreading from cell to cell (Clavaguera et al., 2009; Walker and Jucker, 2024). This contributes to the progression of pathology and neurotoxicity, eventually culminating in widespread neuronal dysfunction and degeneration (Biel et al., 2021). At the molecular level, tau aggregates replicate by templating the conversion of native tau into an amyloid conformation, promoting its accumulation into fibrillar aggregates. For this to occur at the cellular level, seeding-competent tau species (or tau ‘seeds’) must be released from a donor cell and taken up into the cytosol of the neighboring receiving cell in order to come into direct contact with the cytosolic native tau protein (Frost et al., 2009; Sanders et al., 2014; Shrivastava et al., 2019).

In this process, the autophagy–lysosomal pathway (ALP), which is an important clearance route for tau (Caballero et al., 2018), appears to play a central role. Studies have demonstrated impaired ALP function in the brains of tauopathy patients as well as in animal and cell models, showing that the accumulation of abnormal autolysosomal and endolysosomal vesicles correlates with neuronal toxicity (Menzies et al., 2015; Nixon et al., 2005; Hou et al., 2024; Lim et al., 2024). Inhibition of autophagic clearance of tau increases its secretion and spreading (Caballero et al., 2021). In addition, the accumulation of misfolded tau within endolysosomes leads to a destabilization and rupture of these vesicles (Chen et al., 2019; Flavin et al., 2017; Tuck et al., 2022; Dimou et al., 2023; Polanco et al., 2021; Rose et al., 2024). While intact endolysosomes normally restrict tau seeds from reaching cytosolic monomers, membrane rupture allows their escape. Recent studies highlight this escape as a critical rate-limiting step in seeded tau propagation (Chen et al., 2019; Flavin et al., 2017; Tuck et al., 2022; Dimou et al., 2023; Polanco et al., 2021; Rose et al., 2024). Endolysosomal damage not only promotes the propagation of pathological aggregates, but also causes the release of hydrolytic enzymes into the cytosol, leading to cellular damage and death (Kroemer and Jäättelä, 2005). Although recent efforts to identify pathways involved in endolysosomal damage and repair have intensified, many aspects of these processes remain unknown.

**Figure 1.**
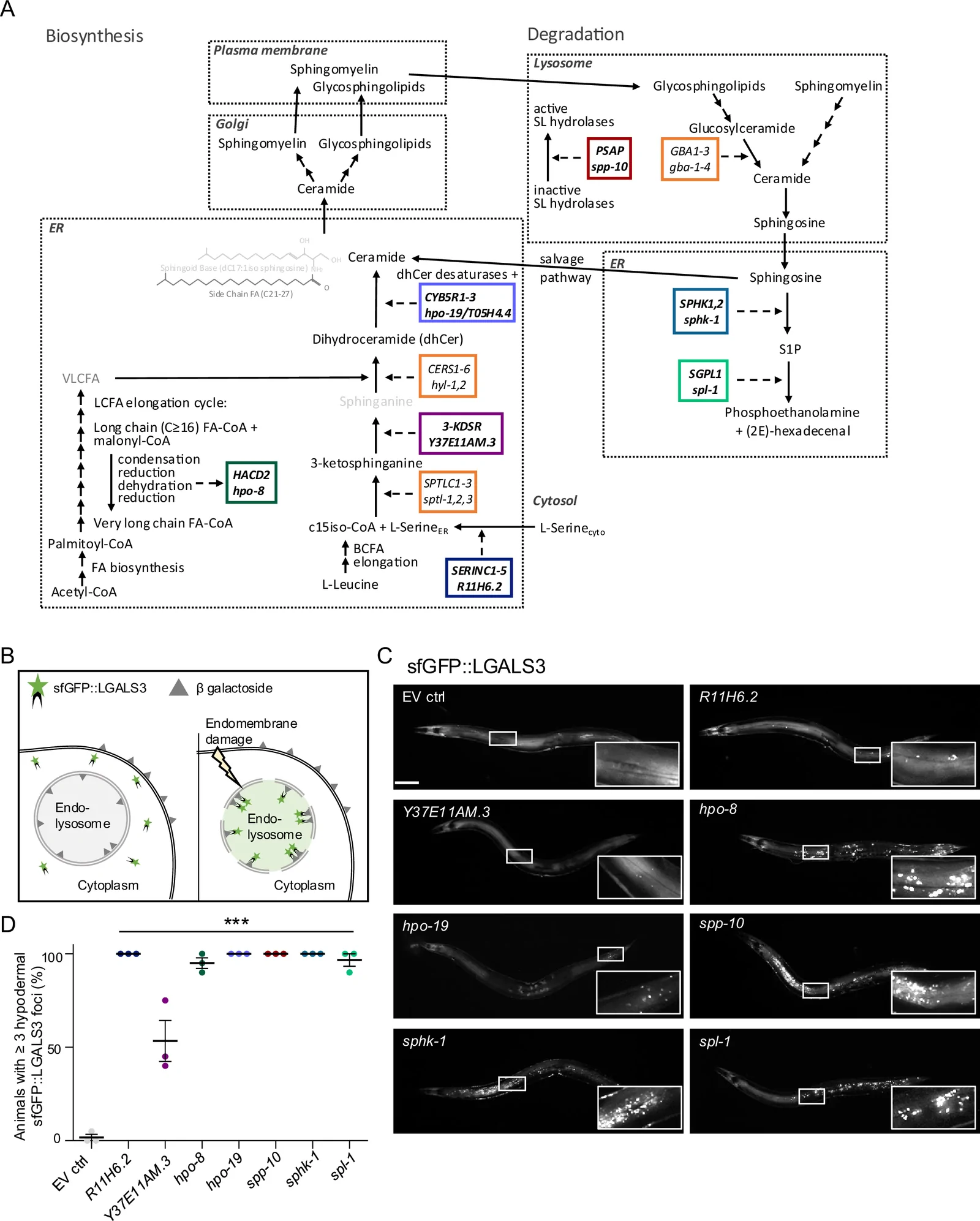
Knockdown of sphingolipid metabolism genes promotes endolysosomal vesicle rupture. (**A**) General overview of sphingolipid metabolism with a particular focus on the genes identified in the genome-wide screen. Sphingolipids constitute a group of amphipathic lipids featuring a polar head group and a sphingoid base backbone that is *N*-acylated with a (very) long-chain fatty acid ((V) LCFA) side chain. In contrast to mammals, where the sphingoid base is conventionally derived from palmitic acid and serine, *C. elegans* sphingolipids usually contain a characteristic C17iso branched chain sphingoid base (Chitwood et al., 1995; Zhang et al., 2011; Zhu et al., 2013). Its synthesis involves the branched chain FA (BCFA) elongation pathway to yield C15iso-CoA, which then condenses with L-serine to form 3-ketosphinganine. This reaction is catalyzed by serine palmitoyltransferase (encoded by *sptl-1*, *-2,* and *-3*). The serine incorporator (SERINC) protein family (encoded by *R11H6.2*) is believed to assist in the incorporation of L-serine into specific membranes. 3-Ketodihydrosphingosine reductase (KDSR, in *C. elegans* predicted to be encoded by *Y37E11AM.3*) then catalyzes the reduction of 3-keto sphinganine to sphinganine. The (V)LCFA side chain is primarily comprised of a straight saturated FA chain, ranging from 20 to 26 carbon atoms in length, with or without hydroxylation (Hänel et al., 2019; Scholz et al., 2021). It can also be derived from BCFAs, such as C15iso and C17iso (Chitwood et al., 1995; Zhang et al., 2011). However, most of the side chain FA moieties originate from palmitoyl-CoA via the canonical de novo FA biosynthesis pathway, involving the sequential addition of C2 moieties from malonyl-CoA through the LCFA elongation cycle (Chitwood et al., 1995; Zhang et al., 2011). Each elongation cycle comprises four reactions (condensation, reduction, dehydration, and reduction), with the third reaction requiring very-long-chain (3*R*)-3-hydroxyacyl-CoA dehydratase (encoded by *hpo-8*) (Zhang et al., 2011). Finally, ceramide synthases (encoded by *hyl-1* and *hyl-2*) catalyze the addition of various acyl side chains to the sphingoid base to yield dihydroceramide, which is then desaturated to ceramide. The latter reaction is catalyzed by dihydroceramide desaturases, which require electrons from NAD(P)H provided by cytochrome b5 reductases (encoded by *hpo-19* and *T05H4.4*). All complex sphingolipids, such as sphingomyelin and glycosphingolipids (including cerebrosides and gangliosides), originate from ceramide. Degradation of complex sphingolipids takes place in the lysosome. Essential for this process are saposins or sphingolipid activator proteins (PSAPs, encoded by *spp-10*), which serve as crucial bridges between the lipid substrate and hydrophilic hydrolases. Glucocerebrosidases (encoded by *gba-1*, *gba-2*, *gba-3*, and *gba-4*) hydrolyze glucosylceramide into ceramide and glucose. Sphingosine may be either recycled and metabolized back into ceramide or phosphorylated by sphingosine kinase (encoded by *sphk-1*) to generate sphingosine-1-phosphate (S1P). S1P lyase (encoded by *spl-1*) irreversibly cleaves S1P into phosphoethanolamine and (2E)-hexadecenal. The *C. elegans* genes identified in the primary screen, along with their human orthologs, are framed with color. Genes identified in subsequent co-RNAi experiments and their human orthologs are framed in gray. (**B**) Schematic of lysosomal rupture detected by the galectin puncta assay. (**C**) Widefield fluorescence images of day 5 (second day of adulthood) animals expressing F3ΔK281::mCherry in touch receptor neurons and hypodermal sfGFP::LGALS3 and RNAi-mediated KD of indicated sphingolipid metabolism genes. Numerous foci are visible indicating lysosomal rupture. Zoomed-in image is indicated in overview image by a white box. Scale bar: 100 µm. (**D**) Mean percentage of day 5 (second day of adulthood) animals positive for lysosomal rupture (defined as three or more sfGFP::LGALS3 foci in the hypodermis). Data shown as means of three technical replicate plates with 17–30 animals per plate ± SEM. Statistical analysis comparing RNAi conditions to the empty vector control (EV ctrl) was done using one-way ANOVA with Dunnett’s post hoc test. ***p < 0.001. Figure 1—source data 1.This Source Data contains raw data for Figure 1.

To identify cellular factors that are critical for the integrity of endolysosomal vesicles, we recently performed an unbiased genome-wide RNA interference (RNAi) screen in *Caenorhabditis elegans* (Sandhof et al., 2025). One of the pathways identified was sphingolipid (SL) metabolism. SLs constitute a large and diverse class of lipids that are involved in various physiological processes (Breslow and Weissman, 2010; Jiménez-Rojo et al., 2020). They consist of two main building blocks, a long-chain base with a serine backbone and an attached long acyl chain. This characteristic chemical structure mediates unique biophysical properties. SL biosynthesis and degradation rely on a specialized enzymatic machinery distinct from that of other lipids (Gault et al., 2010; Figure 1A). Functionally, SLs, along with glycerolipids and sterols, serve as structural components of cell membranes, contributing to the stability and fluidity of membranes and to the organization of microdomains such as lipid rafts. Beyond their structural role, SLs serve as bioactive molecules participating in cellular signaling pathways that control cell growth, differentiation, apoptosis, and intercellular communication (Futerman and Hannun, 2004). Abnormalities in SL metabolism have been observed during aging and in neurodegenerative conditions, including AD, underscoring their potential significance in the pathogenesis of these disorders (Czubowicz et al., 2019; van Echten-Deckert and Walter, 2012). Interestingly, recent evidence suggests that sphingolipid accumulation disturbs the endolysosomal pathway and induces or potentiates endolysosomal membrane rupture (Huotari and Helenius, 2011; Yong et al., 2024); however, the mechanisms underlying membrane destabilization remain unclear.

**Figure 1—figure supplement 1.**
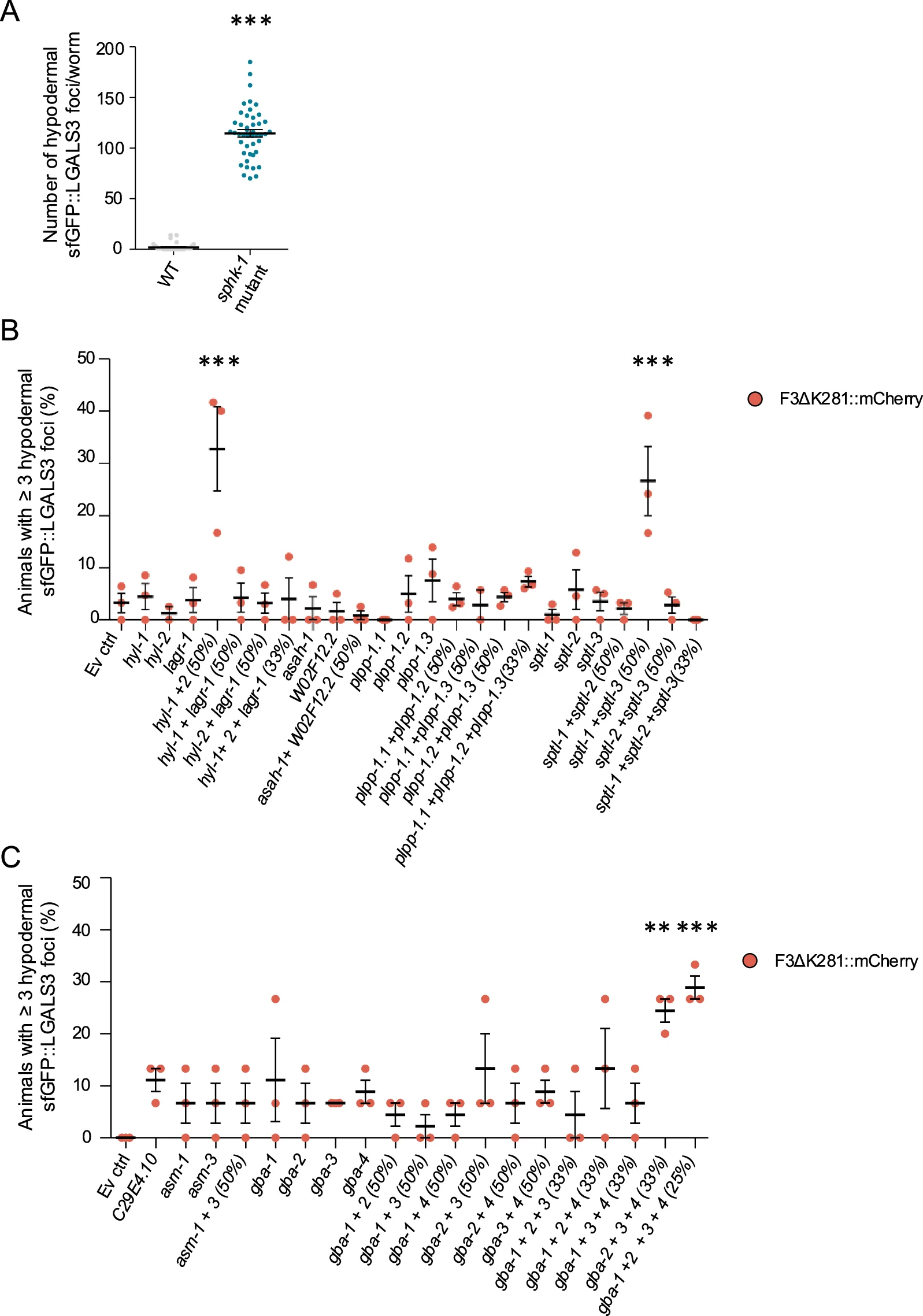
*Sphk-1* and additional sphingolipid metabolic genes affect endolysosomal rupture. (**A**) Genetic validation of the *sphk-1* RNAi phenotype using a *sphk-1* mutant. Quantification of sfGFP::LGALS3 foci per worm on day 5 (second day of adulthood) in wild-type (WT) and *sphk-1* mutant animals. Each dot represents one animal; data are shown as mean ± SEM. *n* = 3 biological repeats with 15 animals per replicate. Statistical analysis was done using an unpaired Student’s *t*-test. (**B, C**) Co-KD of redundant genes involved in SL metabolism identifies additional regulators of endolysosomal rupture. Quantification of the percentage of animals on day 5 (second day of adulthood) expressing F3ΔK281::mCherry in touch receptor neurons with ≥3 hypodermal sfGFP::LGALS3 foci, upon indicated single and co-KDs. Percentages shown next to gene names indicate the relative amount of each RNAi bacterial clone in co-KD conditions. (**B**) Among the genes involved in SL biosynthesis, co-KDs of *sptl-1* and *-3* and *hyl-1* and *-2* significantly induce endolysosomal rupture compared with the empty vector control. (**C**) For genes related to SL degradation, co-KD of *gba-2, -3*, and *-4* or *gba-1,-2*, *-3*, and *-4* resulted in a significant increase in endolysosomal rupture. Data are shown as mean ± SEM. *N* = 45–60 animals from three biological replicates. Statistical analysis was done using one-way ANOVA with Dunnett’s post hoc test, **p < 0.01, ***p < 0.001. Figure 1—figure supplement 1—source data 1.This Source Data contains raw data for Figure 1—figure supplement 1.

Here, we investigated the role of SL metabolism in maintaining endolysosomal vesicle integrity. Our results revealed that silencing of SL metabolism genes led to a marked reduction in membrane fluidity, particularly in membranes of endolysosomal vesicles, thereby increasing their fragility and susceptibility to rupture. The accumulation of aggregated tau had an additive effect and led to a further reduction in endomembrane fluidity, which aggravated the damage to the endolysosomal compartment. Increased membrane rigidity also facilitated seeded tau aggregation. Conversely, improving membrane fluidity through supplementation with polyunsaturated fatty acids (PUFAs) counteracted tau propagation in cells and tau-associated neurotoxicity in *C. elegans*.

This study highlights the interplay between lipid metabolism and proteostasis and provides insights into how perturbations in sphingolipid metabolism contribute to endolysosomal membrane dysfunction and tau-associated phenotypes. In addition, our data offer a potential mechanistic explanation for the beneficial effects of PUFA-rich diets or PUFA supplementation reported in AD patients and suggest that restoring membrane fluidity may help mitigate tau toxicity and disease progression.

## Results

### Disruption of sphingolipid metabolism induces endolysosomal rupture

Alterations in SL metabolism are increasingly recognized in aging and neurodegenerative diseases, including AD; however, it remains unclear whether these changes actively contribute to disease pathology or merely reflect downstream consequences of neurodegeneration. In our previously published unbiased genome-wide RNAi screen in *C. elegans*, we identified SL metabolism as one of the pathways whose disruption compromises endolysosomal membrane integrity (Sandhof et al., 2025). Here, we focused on these SL-related hits to better understand how perturbation of SL metabolism promotes endolysosomal rupture and whether it affects tau-related phenotypes.

To first confirm the SL-related hits under the same assay conditions in which they were originally identified, we used the *C. elegans* reporter strain from our previously published screen (Sandhof et al., 2025; Supplementary file 1). In this strain, endolysosomal membrane damage is monitored in the hypodermis by expression of human galectin-3 fused to superfolder-GFP (sfGFP::LGALS3). The animals also express an aggregation-prone tau fragment fused to mCherry (F3ΔK281::mCherry) in touch receptor neurons, which is transmitted to the hypodermis, as described previously (Sandhof et al., 2025). Under steady-state conditions, sfGFP::LGALS3 remains diffusely distributed throughout the cytosol. Upon endolysosomal damage, luminal β-galactosides become exposed and recruit sfGFP::LGALS3 into visible puncta, providing a sensitive readout of vesicle rupture (Sandhof et al., 2025; Aits et al., 2015; Figure 1B).

Using this reporter system, we confirmed that knockdown (KD) of selected SL-related hits increased sfGFP::LGALS3 foci formation. These hits included the serine incorporator *R11H6.2*, the 3-ketodihydrosphingosine reductase *Y37E11AM.3,* the hydroxyacyl-CoA dehydratase *hpo-8*, the cytochrome b5 reductases *hpo-19* and *T05H4.4,* the saposin *spp-10*, the sphingosine kinase *sphk-1*, and the sphingosine-1-phosphate lyase *spl-1* (Figure 1C, D). As genetic validation independent of RNAi, we tested an available *sphk-1* mutant strain, which also showed a robust increase in hypodermal sfGFP::LGALS3 foci (Figure 1—figure supplement 1A). These data support the conclusion that genetic perturbation of sphingolipid metabolism compromises endolysosomal integrity.

The screen identified distinct steps in sphingolipid metabolism catalyzed by non-redundant genes or targeted by a single RNAi clone, as in the case of *hpo-19*/*T05H4.4*, which are both depleted by *hpo-19* RNAi (Figure 1A). However, other steps in this pathway, which are mediated by two or more proteins, may have been missed in the screen. Indeed, simultaneous KD of selected redundant genes using co-RNAi revealed additional genes functioning in de novo sphingolipid biosynthesis that also induced endolysosomal rupture, including the serine palmitoyltransferases *sptl-1* and *-3* and the ceramide synthases *hyl-1* and *-2* (Figure 1—figure supplement 1B). In addition, the co-KD of all four glucocerebrosidases *gba-1–4* or only *gba-2–4* also triggered endolysosomal rupture (Figure 1—figure supplement 1C).

Together, these results indicate that perturbing SL metabolism at multiple steps can compromise endolysosomal integrity, highlighting the tight metabolic balance required to maintain the stability of the endolysosomal limiting membrane. Because KD efficiency was not assessed for the individual RNAi clones or co-RNAi combinations, these experiments do not allow comparison of relative RNAi strength or inference of the relative importance of individual genes. Thus, the conclusions drawn from these RNAi experiments are qualitative: specific single or combined KDs can promote endolysosomal rupture, whereas the absence of a detectable phenotype after RNAi cannot exclude gene involvement, as KD may have been insufficient.

### Disruption of sphingolipid metabolism reduces endolysosomal membrane fluidity in *C. elegans*

SLs are important components of eukaryotic cell membranes, and genetic modulation of SL metabolism likely affects the lipid composition and biophysical properties of membranes. Similar to their mammalian isoforms, the *N*-acyl chains of *C. elegans* SLs contain predominantly long and saturated fatty acid chains (Hänel et al., 2019; Cheng et al., 2019), which tend to pack tightly within the lipid bilayer. As such, a higher proportion of SLs generally reduces membrane fluidity, leading to increased order, rigidity, or viscosity (D’Auria and Bongarzone, 2016). Consequently, KD of the hits involved in SL degradation should lead to SL accumulation and reduced membrane fluidity, while KD of the hits involved in SL biosynthesis should have the opposite effect.

**Figure 2.**
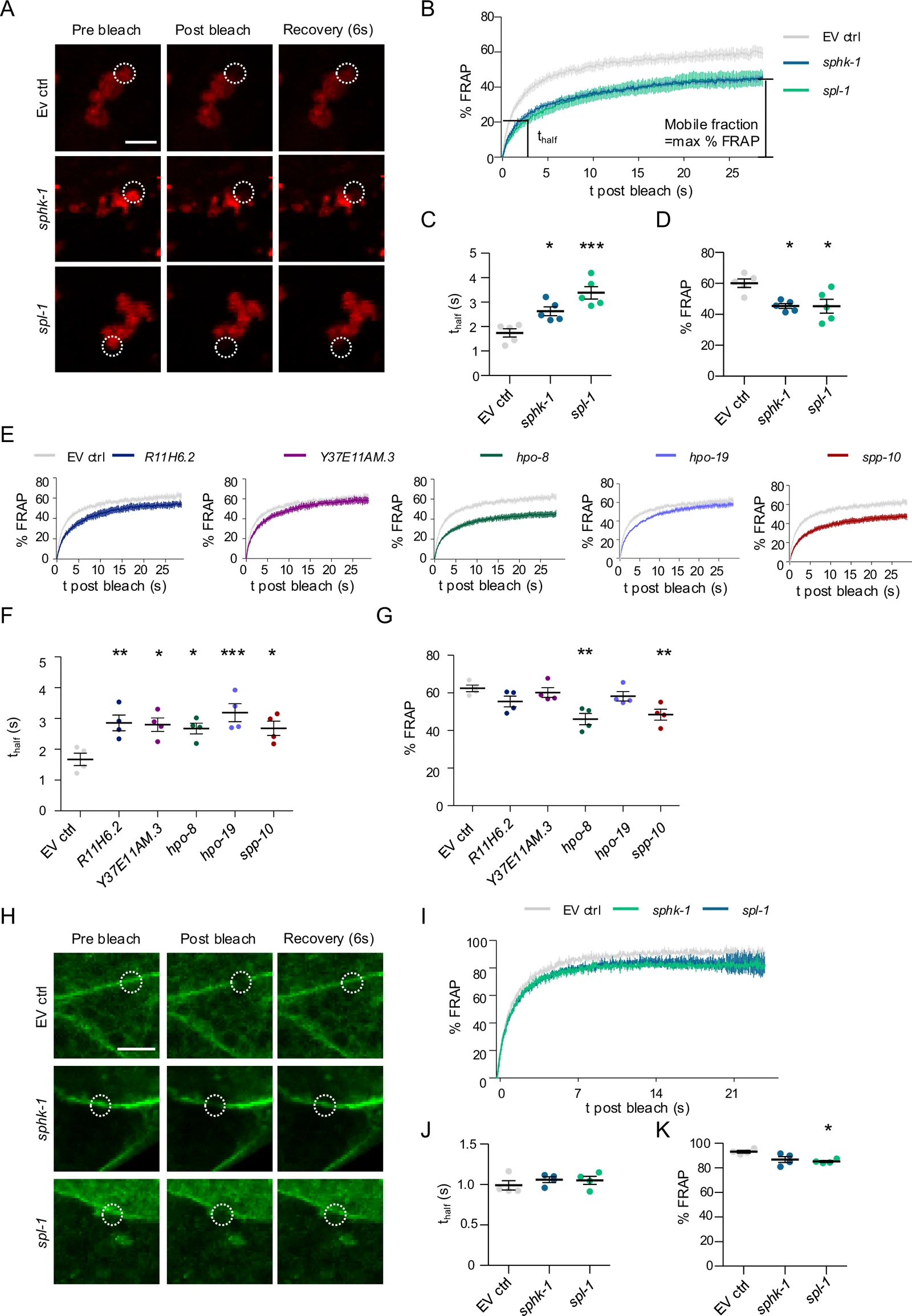
Knockdown of genes involved in sphingolipid metabolism decreases lysosomal membrane fluidity. (**A**) Representative confocal single plane images from an FRAP experiment in day 5 (second day of adulthood) animals expressing a mCherry-tagged Lysosomal Lysine/Arginine Transporter 1 (LAAT-1::mCherry) in the hypodermis grown either on empty vector control (EV ctrl), *spl-1* or *sphk-1* RNAi plates. Dashed circles outline the bleach spots. Scale bar = 10 µm. (**B**) Combined FRAP curves of LAAT-1::mCherry in hypodermal lysosomal membranes. Curves are normalized to the pre-bleach intensity as 100% and the first post-bleach intensity as 0%. Increase (**C**) in the mean time until half of the maximal signal is recovered (*t*_half_) and decrease (**D**) in the maximal % recoverable fluorescence values upon KD of *sphk-1* and *spl-1* indicate a reduction in lysosomal membrane fluidity. (**E**) FRAP curves of LAAT-1::mCherry in hypodermal lysosomal membranes of animals grown either on empty vector or the indicated RNAi plates. Curves are normalized to the pre-bleach intensity set as 100% and the first post-bleach intensity as 0%. (**F**) Mean *t*_half_ upon KD of sphingolipid metabolism genes. (**G**) Maximal % recoverable fluorescence values upon KD of sphingolipid metabolism genes. (**H**) Representative confocal single plane images from an FRAP experiment in animals expressing prenylated GFP for lipid membrane anchorage in the intestine. Scale bar = 5 µm. (**I**) Combined FRAP curves of prenylated GFP enriched on the intestinal plasma membrane of animals grown either on empty vector, *sphk-1* or *spl-1* RNAi plates. Curves are normalized to the pre-bleach intensity as 100% and the first post-bleach intensity as 0%. Mean *t*_half_ (**J**) and maximal % recoverable fluorescence values (**K**). Data represented as means ± SEM of 5–12 FRAP measurements per condition in animals on day 5 (second day of adulthood) collected in five biological replicates. Statistical analysis comparing RNAi conditions to the empty vector control was done using one-way ANOVA with Dunnett’s post hoc test. n.s.: not significant, *p < 0.05, **p < 0.01, ***p < 0.001. Figure 2—source data 1.This Source Data contains raw data for Figure 2.

To evaluate the membrane fluidity of endolysosomal membranes, fluorescence recovery after photobleaching (FRAP) was employed on *C. elegans* that express the Lysine/Arginine Transporter 1 (LAAT-1) tagged with mCherry, which localizes to the lysosomal membrane (Liu et al., 2012). FRAP enables the assessment of the lateral mobility of LAAT-1 within the lysosomal membrane, thereby indirectly measuring its fluidity. We focused our analysis on the hypodermis, where we expressed the sfGFP::LGALS3 reporter and observed endolysosomal rupture following KD of the SL metabolism genes (Figure 1C, D). Interestingly, KD of all of our hits significantly increased the time required to recover half of the maximum fluorescence intensity (*t*_half_), indicating less fluid lysosomal membrane in the hypodermis (Figure 2A–C, E, F). With some gene KDs, we also observed a decrease in the maximal recovered signal after bleaching (Figure 2D, G). The decrease in lysosomal membrane fluidity was not specific to the hypodermis as the KD of *spl-1* also increased the *t*_half_ of LAAT-1::mCherry in intestinal lysosomes (Figure 2—figure supplement 1A–D). To discern whether the detrimental effect of KD on membrane fluidity is confined to lysosomal membranes or extends to other cellular membranes, we utilized a *C. elegans* strain expressing a plasma membrane-anchored GFP (Mörck et al., 2009). Intriguingly, this reporter showed no change in the *t*_half_ with any KD and only *spl-1* KD led to a decrease in the maximal recovered signal (Figure 2H–K, Figure 2—figure supplement 1E–G). Hence, the endolysosomal membrane seems to be particularly sensitive to a disruption of SL metabolism in contrast to the plasma membrane. Perturbing genes involved in both SL synthesis and degradation reduces the fluidity of the endolysosomal membrane and makes it more susceptible to rupture.

**Figure 2—figure supplement 1.**
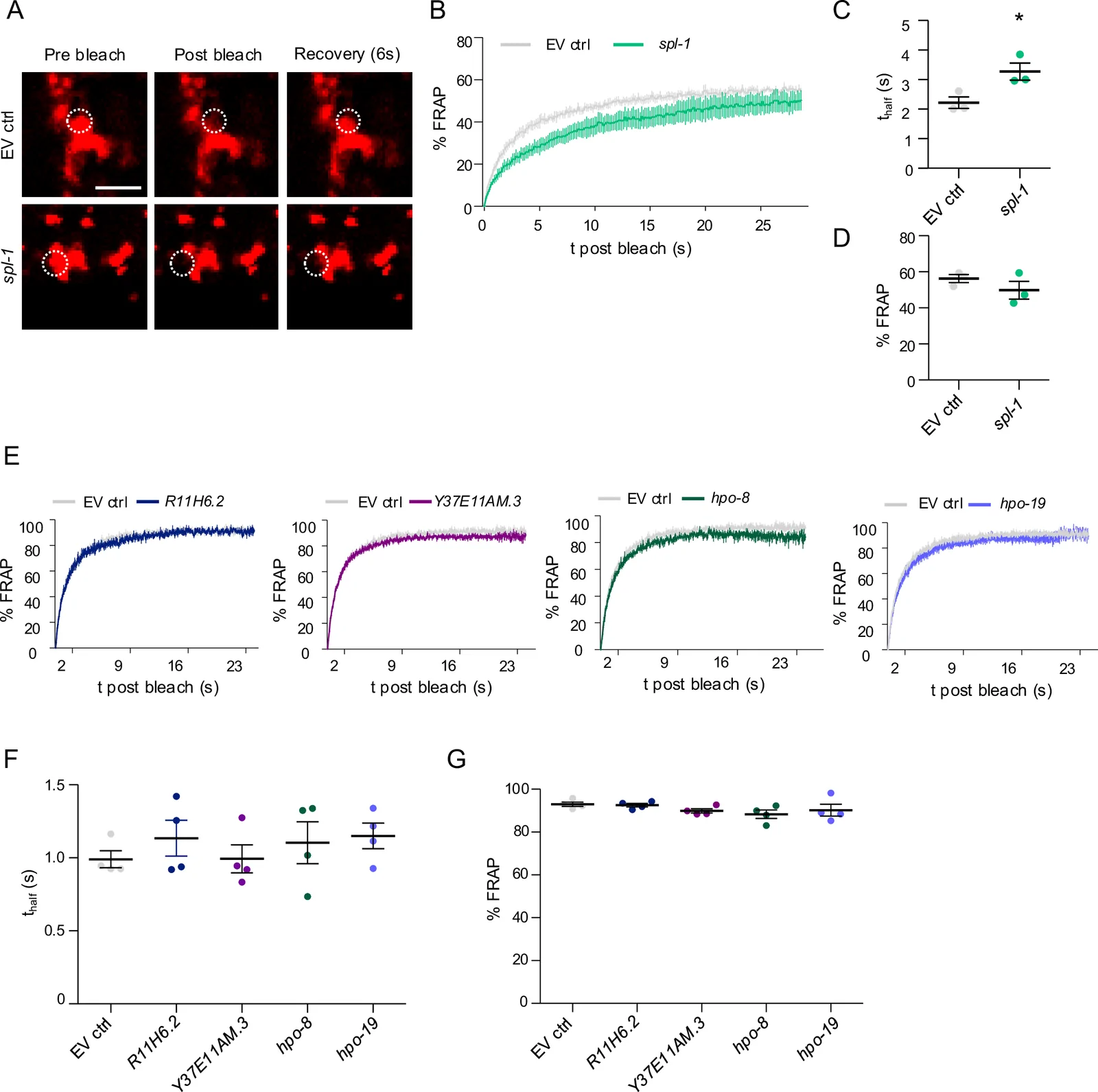
Disruption of SL metabolism decreases lysosomal membrane fluidity. (**A**) Representative confocal single plane images from an FRAP experiment in animals on day 5 (second day of adulthood) expressing a mCherry-tagged Lysosomal Lysine/Arginine Transporter 1 (LAAT-1::mCherry) in the intestine grown either on empty vector or *spl-1* RNAi plates. Dashed circles outline the bleach spots. Scale bar = 5 µm. (**B**) Combined FRAP curves of LAAT-1::mCherry in intestinal lysosomes of animals on day 5 (second day of adulthood) grown either on empty vector or *spl-1* RNAi plates. Curves are normalized to the pre-bleach intensity as 100% and the first post-bleach intensity as 0%. Mean *t*_half_ (**C**) and maximal % recoverable fluorescence values (**D**) calculated from the LAAT-1::mCherry FRAP experiments. (**E**) FRAP curves of prenylated GFP enriched on the intestinal plasma membrane of animals on day 5 (second day of adulthood) grown either on empty vector or the indicated RNAi plates. Curves are normalized to the pre-bleach intensity set as 100% and the first post-bleach intensity as 0%. Mean *t*_half_ (**F**) and maximal % recoverable fluorescence values (**G**) calculated from the GFP FRAP experiments. Data, including FRAP curves, represented as means ± SEM from 5–12 FRAP measurements on day 5 animals (second day of adulthood). Statistical analysis comparing RNAi conditions to the empty vector control was done using one-way ANOVA with Dunnett’s post hoc test. *p < 0.05. Figure 2—figure supplement 1—source data 1.This Source Data contains raw data for Figure 2—figure supplement 1.

### KD of SPHK2 increases endolysosomal membrane rigidity in human cells

**Figure 3.**
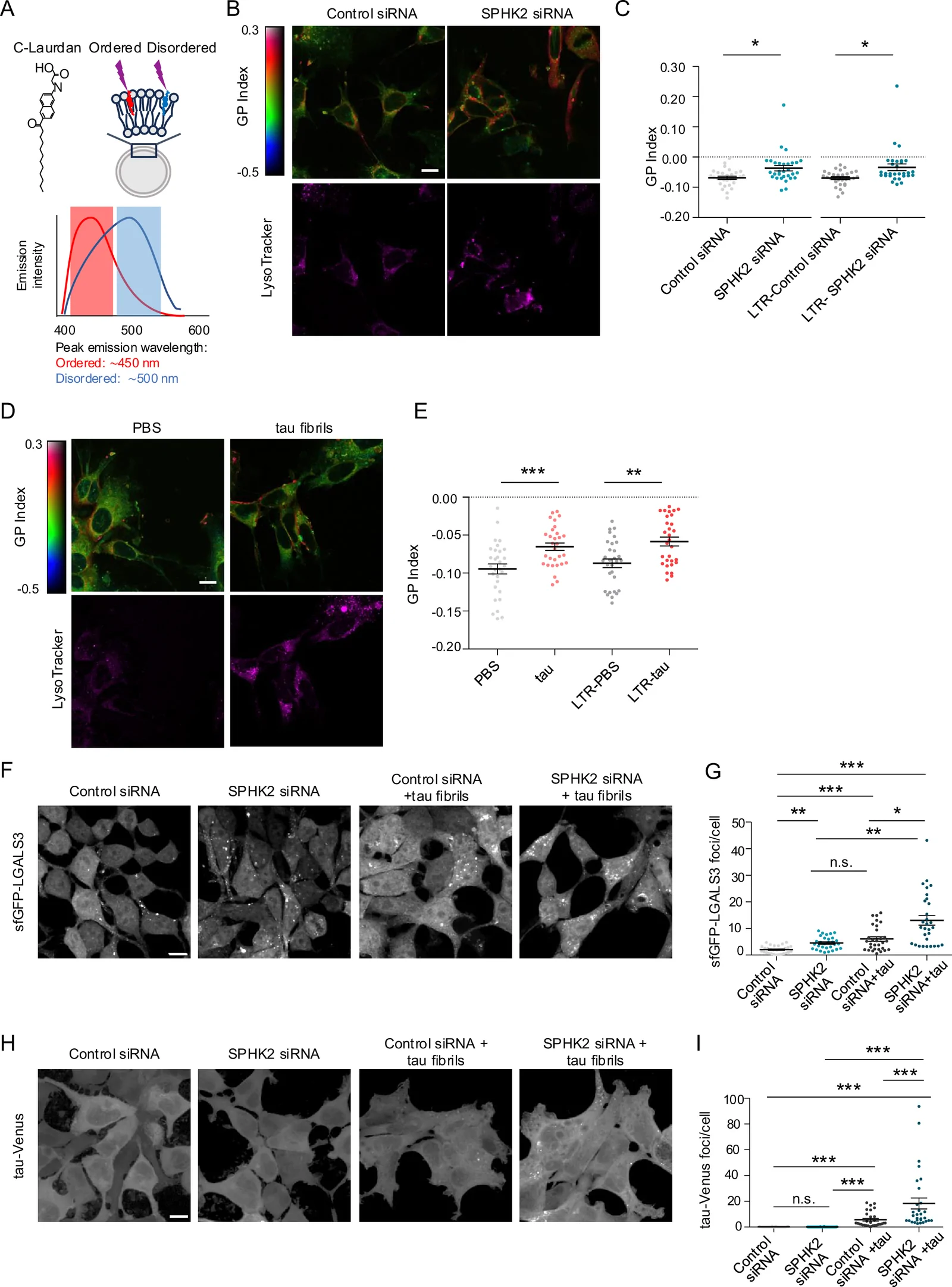
KD of SPHK2 and aggregated tau increase membrane rigidity leading to lysosomal rupture. (**A**) Scheme of the fluorescence properties of C-Laurdan. The dye is excited at 405 nm and exhibits peak emission at 450 nm (red) in ordered membrane phases and ~500 nm in the disordered phase (blue). Two-channel acquisition is conducted in the wavelength bands indicated by shaded boxes. (**B**) Upper panels: Pseudo-colored images of SH-SY5Y cells transfected with control or SPHK2 siRNA showing the C-Laurdan GP Index at each pixel position. Lower panels show LysoTracker staining. Scale bar = 10 µm. (**C**) Quantification of GP values in SH-SY5Y cells after transfection with control or SPHK2 siRNA. GP values were measured across the whole cell (left) or restricted to LysoTracker-positive (LTR) regions (right). Statistical analysis was conducted using a two-way mixed-model ANOVA, followed by pairwise comparisons of estimated marginal means with Sidak correction for multiple comparisons. *n* = 3 independent experiments, with 10 images analyzed per experiment. (**D**) Upper panel: Pseudo-colored images of SH-SY5Y cells exposed to PBS control or 1N4R tau fibrils showing the C-Laurdan GP Index at each pixel position. Lower panels show LysoTracker staining. Scale bar = 10 µm. (**E**) Quantification of GP values in SH-SY5Y cells exposed to PBS control or 1N4R tau fibrils. GP values were measured across the whole cell (left) or restricted to LysoTracker-positive (LTR) regions (right). Statistical analysis was conducted using a two-way mixed-model ANOVA followed by pairwise comparisons of estimated marginal means with Sidak correction for multiple comparisons. *n* = 3 independent experiments, with 10 images analyzed per experiment. (**F**) Maximum intensity projection of confocal z-stacks of HEK293T cells expressing sfGFP-LGALS3 upon treatment with control or SPHK2 siRNA, exposed to PBS control or 1N4R tau fibrils. Scale bar = 10 µm. (**G**) Quantification of sfGFP-LGALS3 foci per cell in HEK293T cells upon treatment with control or SPHK2 siRNA, exposed to PBS control or 1N4R tau fibrils. Data represent the mean number of foci per cell. Statistical analysis was done using Kruskal–Wallis with a Dunn’s post hoc test. *n* = 3 independent experiments, with 10 images analyzed per experiment. (**H**) Maximum intensity projection of confocal z-stacks of a P301S tau-Venus biosensor cell line upon treatment with control or SPHK2 siRNA with and without exposure to 1N4R tau fibrils. Scale bar = 10 µm. (**I**) Quantification of tau-Venus foci upon treatment with control or SPHK2 siRNA with and without exposure to 1N4R tau fibrils. Data were analyzed by a two-way mixed-model ANOVA, followed by pairwise comparisons of estimated marginal means with Sidak correction. *n* = 3 independent experiments, with 10 images analyzed per experiment. n.s.: not significant, *p < 0.05, **p < 0.01, ***p < 0.001. Figure 3—source data 1.This Source Data contains raw data for Figure 3.

Having established in *C. elegans* that disruption of SL metabolism reduces endolysosomal membrane fluidity, we next asked whether this relationship is conserved in human cells. To this end, we employed SH-SY5Y human neuroblastoma cells and assessed membrane fluidity using C-Laurdan dye (Figure 3A). This approach leverages the unique properties of C-Laurdan, which emits fluorescent light of varying wavelengths in response to changes within the phospholipid bilayer, particularly reflective of membrane fluidity (Owen et al., 2011; Barucha-Kraszewska et al., 2013). The shift in emission profile between liquid-disordered and liquid-ordered phases allows a quantitative assessment of the membrane order by calculating the ratiometric relationship of the fluorescence intensity recorded in two spectral channels, known as a generalized polarization (GP) value (Owen et al., 2011; Figure 3A). Decreasing GP values are indicative of increasingly fluid membrane while increasing GP values denote increased rigidity. Of note, we exposed the cells to C-Laurdan for an extended time of 2 hr (instead of 30 min as recommended in the original protocol), to promote internalization of the dye and thus increase the staining of endolysosomal membranes relative to the plasma membrane (Owen et al., 2011).

**Figure 3—figure supplement 1.**
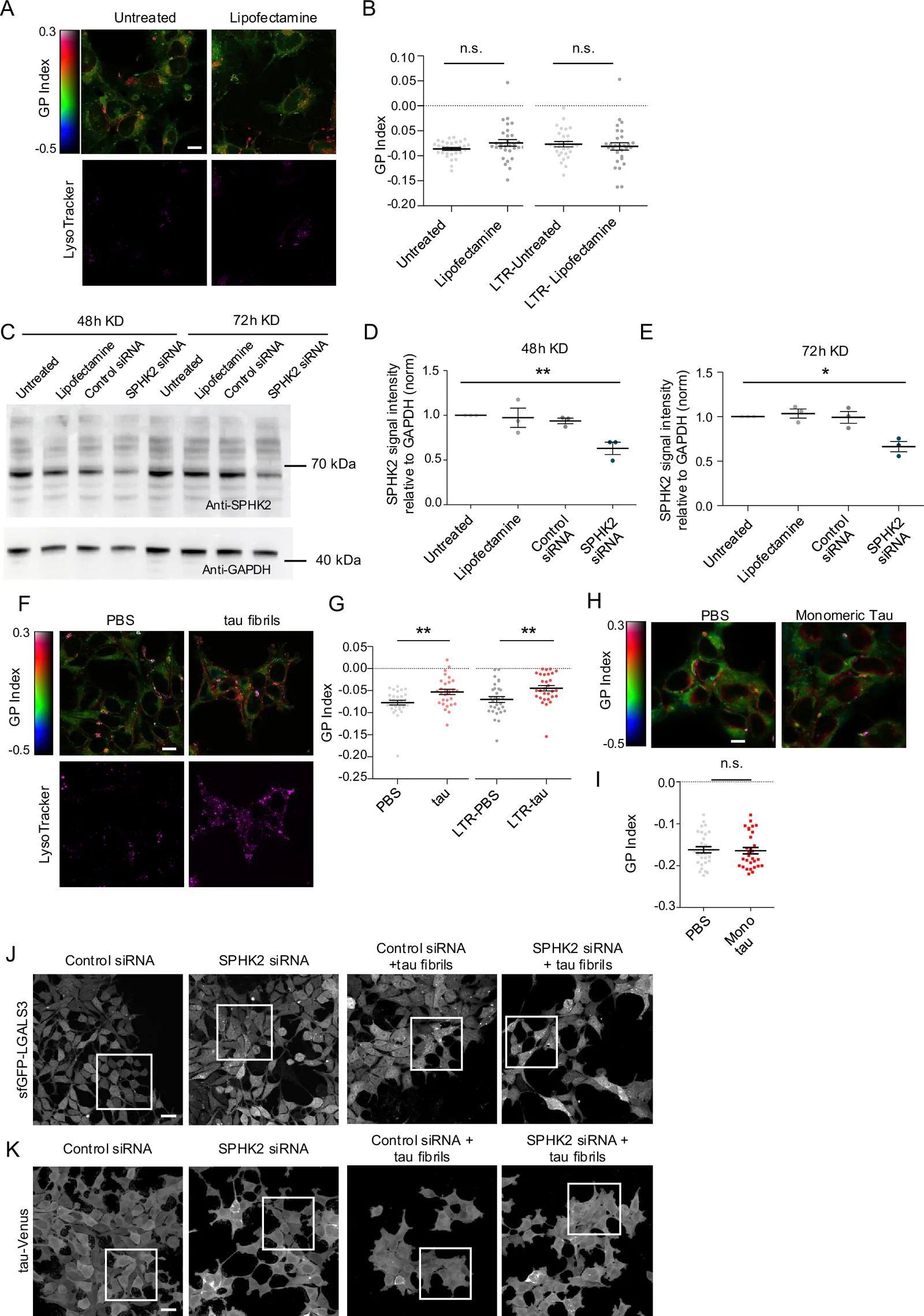
KD of SPHK2 and exposure to fibrillar tau decrease membrane fluidity in human cells. (**A**) Upper panels: pseudo-colored images of SH-SY5Y cells untreated or transfected with Lipofectamine showing the C-Laurdan GP Index. Lower panels: LysoTracker staining. Scale bar = 10 µm. (**B**) Quantification of GP values in SH-SY5Y cells that were untreated or treated with Lipofectamine alone. GP values were measured either across the whole cell (left) or specifically within LysoTracker-positive (LTR) regions (right). No significant differences were observed (see also Figure 3C). Statistical analysis was performed using a two-way mixed-model ANOVA followed by pairwise comparisons of estimated marginal means with Sidak correction for multiple comparisons. *n* = 3 independent experiments, with 10 images analyzed per experiment. (**C**) Western blot analysis of total cell lysates from SH-SY5Y cells after 48 or 72 hr of no treatment, Lipofectamine treatment alone, or transfection with control or SPHK2 siRNA using Lipofectamine. SPHK2 and GAPDH protein levels were assessed using anti-SPHK2 and anti-GAPDH antibodies, respectively (see methods section). Quantification of SPHK2 protein levels relative to GAPDH after 48 hr (**D**) or 72 hr (**E**) of indicated controls or SPHK2 siRNA treatment. Each dot represents an independent experiment, with lines indicating mean ± SEM. Statistical analysis was performed using one-way ANOVA followed by Dunnett’s post hoc test. (**F**) Upper panels: pseudo-colored images of HEK293T cells exposed to PBS control or 1N4R tau fibrils showing the C-Laurdan GP Index. Lower panels: LysoTracker staining. Scale bar = 10 µm. (**G**) Quantification of GP values in HEK293T cells exposed to PBS control or 1N4R tau fibrils. Increased GP values indicate increased membrane rigidity following tau fibril treatment. Statistical analysis was performed using a two-way mixed-model ANOVA followed by pairwise comparisons of estimated marginal means with Sidak correction for multiple comparisons. *n* = 3 independent experiments, with 10 images analyzed per experiment. (**H**) Pseudo-colored images of HEK293T cells exposed to PBS control or monomeric 1N4R tau displaying the C-Laurdan GP Index. Scale bar = 10 µm. (**I**) Quantification of GP values in HEK293T cells exposed to PBS control or monomeric 1N4R tau. Statistical analysis was performed using Student’s *t*-test. *n* = 3 independent experiments, with 10 images analyzed per experiment. (**J**) Max. intensity projection of confocal z-stacks of HEK293T cells expressing sfGFP-LGALS3 following control or SPHK2 siRNA treatment, with or without 1N4R tau fibril seeding. White box indicates the zoomed in section depicted in Figure 3F. Scale bar = 20 µm. (**K**) Max. intensity projection of confocal z-stacks of P301S mutant tau-Venus biosensor cell line following control or SPHK2 siRNA treatment, with or without 1N4R tau fibril seeding. White box indicates the zoomed in section depicted in Figure 3H. Scale bar = 20 µm. n.s.: not significant, *p < 0.05, **p < 0.01. Figure 3—figure supplement 1—source data 1.This Source Data contains raw data for Figure 3—figure supplement 1. Figure 3—figure supplement 1—source data 2.This Source Data contains Original membranes corresponding to Figure 3, panel C. Figure 3—figure supplement 1—source data 3.This Source Data contains Original membranes corresponding to Figure 3, panel C.

Upon KD of *SPHK2*, one of the human homologs of *C. elegans sphk-1*, the C-Laurdan fluorescence GP Index increased significantly, indicating increased membrane rigidity (Figure 3B, C). Lipofectamine treatment alone did not alter GP values (Figure 3—figure supplement 1A, B), and SPHK2 KD was confirmed by immunoblotting after 48 and 72 hr (Figure 3—figure supplement 1C–E). To determine whether SPHK2 KD affects lysosomal membrane fluidity, we combined C-Laurdan staining with LysoTracker staining and selectively analyzed LysoTracker-positive regions. SPHK2 KD resulted in a pronounced increase in GP values within LysoTracker-positive compartments, demonstrating increased membrane rigidity at lysosomes (Figure 3C). Taken together, FRAP and C-Laurdan analyses revealed that disruption of SL metabolism leads to more ordered and rigid endolysosomal membranes in both *C. elegans* and human cells.

### Fibrillar tau and SPHK2 KD act in concert to exacerbate endolysosomal damage and seeded tau aggregation

Given that SPHK2 KD increased endolysosomal membrane rigidity and can compromise endolysosomal integrity, we next asked whether aggregated tau similarly alters membrane properties. Since fibrillar tau is known to induce endolysosomal rupture (Flavin et al., 2017), we hypothesized that tau-induced membrane damage may also involve increased membrane rigidity. Treatment of SH-SY5Y and HEK293T cells with recombinant 1N4R tau fibrils led to a marked increase in membrane rigidity, as indicated by elevated GP values (Figure 3D, E, Figure 3—figure supplement 1F, G). This increase was also observed in LysoTracker-positive compartments, indicating increased rigidity of lysosomal membranes. Importantly, this effect was specific to fibrillar tau, as monomeric tau did not alter membrane fluidity (Figure 3—figure supplement 1H, I). These findings suggest that aggregated tau, like SPHK2 KD, promotes membrane rigidification, providing a potential mechanism by which tau fibrils may contribute to endolysosomal rupture.

We therefore asked whether SPHK2 KD further enhances tau-fibril-induced endolysosomal damage. To test this, we used a previously established HEK293T cell line stably expressing sfGFP-LGALS3 and monitored endolysosomal rupture by galectin puncta formation (Sandhof et al., 2025; Aits et al., 2015). Cells were treated with recombinant 1N4R tau fibrils, either alone or in combination with siRNA-mediated SPHK2 KD. Both tau fibrils and SPHK2 KD alone induced endolysosomal rupture, as evidenced by increased sfGFP-LGALS3 puncta formation (Figure 3F, G, Figure 3—figure supplement 1J). While SPHK2 KD alone significantly increased galectin puncta above the matched control, its effect was more modest than in our previous CRISPR inhibition-based analysis (Sandhof et al., 2025). This difference likely stems from the earlier readout required for the combined siRNA/tau fibril assay, when transient Lipofectamine-associated effects still increased the control background. Importantly, SPHK2 KD further enhanced tau fibril-induced sfGFP-LGALS3 foci formation, indicating that SL disruption compromises endolysosomal integrity and sensitizes endolysosomal membranes to tau fibril-induced damage in human cells.

Because endolysosomal rupture facilitates the escape of luminal contents, including tau seeds, into the cytosol, we next asked whether membrane perturbation by SPHK2 KD influences seeded tau aggregation. Using a HEK biosensor cell line expressing Venus-tagged full-length P301S mutant 0N4R tau (tau-Venus) (McEwan et al., 2017; Nachman et al., 2020), we found that SPHK2 KD alone did not induce tau aggregation, as no increase in tau-Venus foci was observed (Figure 3H, I, Figure 3—figure supplement 1K). However, upon addition of recombinant tau fibrils, SPHK2 KD significantly increased tau-Venus foci formation. Thus, under the conditions tested here, disruption of SL metabolism alone is not sufficient to initiate detectable tau aggregation. Rather, it seems to facilitate the escape of tau seeds from the endolysosomal compartment into the cytosol by compromising endolysosomal membrane integrity.

### Tau transmission sensitizes endolysosomal membranes to sphingolipid perturbations in vivo

**Figure 4.**
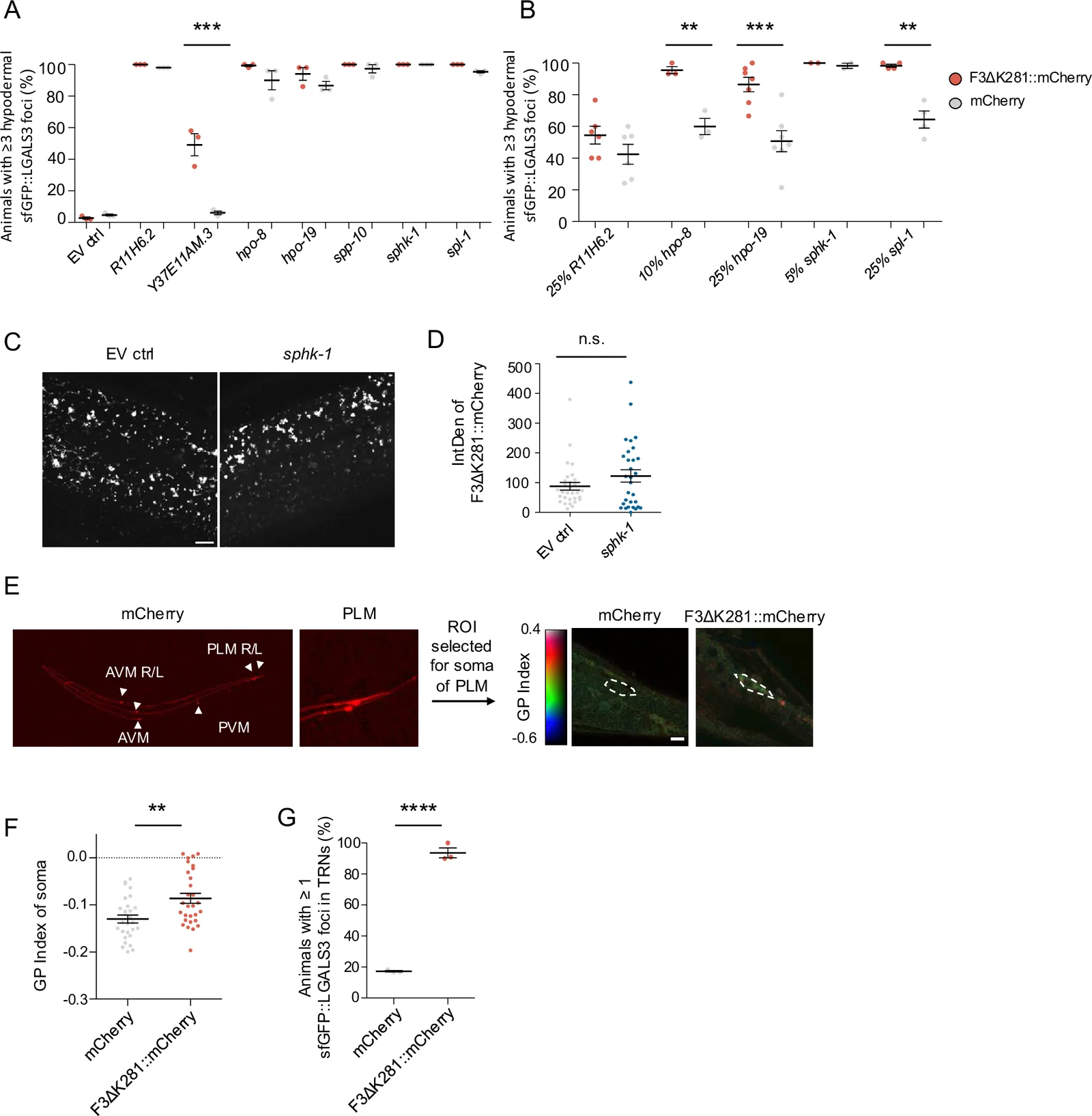
Aggregated tau promotes endolysosomal rupture. (**A, B**) Quantification of the percentage of animals with ≥3 hypodermal sfGFP::LGALS3 foci, upon expression of either F3ΔK281::mCherry (red) or mCherry control (gray) in touch receptor neurons and KD of the indicated genes on day 5 (second day of adulthood) (**A**). Quantification of endolysosomal rupture under sub-saturating RNAi conditions. RNAi cultures were diluted with the empty vector (EV) control bacteria to reduce the KD strength (**B**). Data represented as mean ± SEM. *n* = 3–7 independent experiments with 40–50 (**A**) or 20–30 (**B**) animals analyzed per experiment. Since even a dilution down to 5% of the *sphk-1* RNAi resulted in 100% of animals being scored as positive in two independent replicates this condition was not repeated further. Statistical analysis comparing mCherry to F3ΔK281::mCherry under individual RNAi conditions was done using two-way ANOVA with Sidak’s post hoc test. **p < 0.01, ***p < 0.001. (**C**) Representative images of hypodermal F3ΔK281::mCherry signal in EV control or *sphk-1* RNAi treated animals on day 5 (second day of adulthood). Scale bar = 10 µm. (**D**) Quantification of hypodermal F3ΔK281::mCherry fluorescence intensity (integrated density, IntDen) in EV control or *sphk-1* RNAi treated animals on day 5 (second day of adulthood), indicating *sphk-1* KD does not alter tau transmission levels. Statistical analysis was done using Student’s *t*-test. *n* = 3 independent experiments with 35 animals analyzed in total. (**E**) C-Laurdan staining of animals expressing F3ΔK281::mCherry or mCherry in touch receptor neurons on day 4 (first day of adulthood). The mCherry signal was used to select a region of interest (ROI) around the soma of the posterior touch receptor neurons (PLM) to determine the GP value. Scale bar = 10 µm. (**F**) Quantification of GP values in animals expressing F3ΔK281::mCherry compared to mCherry on day 4 (first day of adulthood). Statistical analysis was done using a Student’s *t*-test. *n* = 4 independent experiments, with at least 28 animals analyzed in total. (**G**) Quantification of the percentage of animals expressing mCherry or F3ΔK281::mCherry in touch receptor neurons with ≥1 sfGFP::LGALS3 puncta in touch receptor neurons on day 5 (second day of adulthood). Each dot represents an independent experiment, and lines indicate the mean ± SEM. *n* = 3 independent experiments, with 10–14 animals per experiment. Statistical analysis was done using a Student’s *t*-test. n.s.: not significant, *p < 0.05, **p < 0.01, ***p < 0.001, ****p < 0.0001. Figure 4—source data 1.This Source Data contains raw data for Figure 4.

To determine how tau transmission and SL perturbations interact to affect endolysosomal integrity in vivo, we returned to *C. elegans*. We compared animals expressing F3ΔK281::mCherry in touch receptor neurons, from where it is transmitted to the hypodermis, with matched controls expressing mCherry alone in the same neurons (Sandhof et al., 2025). In both strains, sfGFP::LGALS3 is expressed in the hypodermis to monitor endolysosomal membrane damage (Sandhof et al., 2025). KD of Y37E11AM.3 alone did not increase hypodermal sfGFP::LGALS3 puncta compared to EV control RNAi, whereas the presence of transmitted F3ΔK281::mCherry significantly increased galectin puncta (Figure 4A). In contrast, KD of the remaining SL-related hits resulted in nearly all animals displaying hypodermal sfGFP::LGALS3 foci in both genetic backgrounds. This suggests that strong disruption of SL metabolism is sufficient to overwhelm endolysosomal integrity independently of tau.

To determine whether tau-dependent effects were masked by the strength of RNAi, we titrated RNAi bacterial concentrations to achieve sub-saturating KD conditions. Under these conditions, KD of *spl-1*, *hpo-8*, and *hpo-19* induced endolysosomal damage that was significantly exacerbated in F3ΔK281::mCherry animals compared to mCherry controls (Figure 4B). In contrast, dilution of *R11H6.2* RNAi did not reveal tau-dependent differences, and *sphk-1* KD remained fully penetrant even at high dilution. Importantly, RNAi-mediated knockdown of *sphk-1* did not alter hypodermal F3ΔK281::mCherry levels, arguing that the enhanced rupture phenotype is not due to increased tau transmission (Figure 4C, D).

To assess whether tau directly alters membrane properties in vivo, we measured membrane fluidity in the somas of the posterior touch receptor (PLM) neurons using C-Laurdan dye. Expression of the tau fragment led to a decrease in membrane fluidity compared to mCherry controls (Figure 4E, F), indicating that tau promotes membrane rigidification in PLM neurons, consistent with our observations in human cells (Figure 3D, E). In addition, expression of sfGFP-LGALS3 in touch receptor neurons revealed increased galectin puncta in animals expressing F3ΔK281::mCherry compared to mCherry controls, indicating elevated endolysosomal rupture in neurons (Figure 4G).

These findings suggest that perturbation of SL metabolism and tau accumulation both increase endolysosomal membrane rigidity and make vesicles more prone to rupture, with additive effects when both perturbations occur simultaneously.

### Increasing membrane fluidity reduces aggregated tau-mediated endolysosomal damage, seeded aggregation, and neurotoxicity

SL metabolism can influence cellular physiology through multiple mechanisms beyond its effects on membrane fluidity. To test more directly whether altered membrane fluidity contributes to aggregated tau-induced endolysosomal damage and propagation, we used fatty acid supplementation as an independent approach to modulate lipid packing. Saturated fatty acids such as palmitic acid (PA) promote tight lipid packing and increase membrane rigidity, whereas unsaturated fatty acids contain one or more double bonds that introduce kinks into their hydrocarbon chains, resulting in looser lipid packing and increased membrane fluidity.

We first increased membrane rigidity with PA. PA treatment increased membrane rigidity in SH-SY5Y and HEK293T cells (Figure 5—figure supplement 1A–F), exacerbated tau fibril-induced sfGFP-LGALS3 foci formation (Figure 5—figure supplement 1G, H), and enhanced tau-Venus aggregation after exposure to tau fibrils (Figure 5—figure supplement 1I, J). These findings support a model in which increased membrane rigidity promotes endolysosomal damage and facilitates tau propagation.

**Figure 5.**
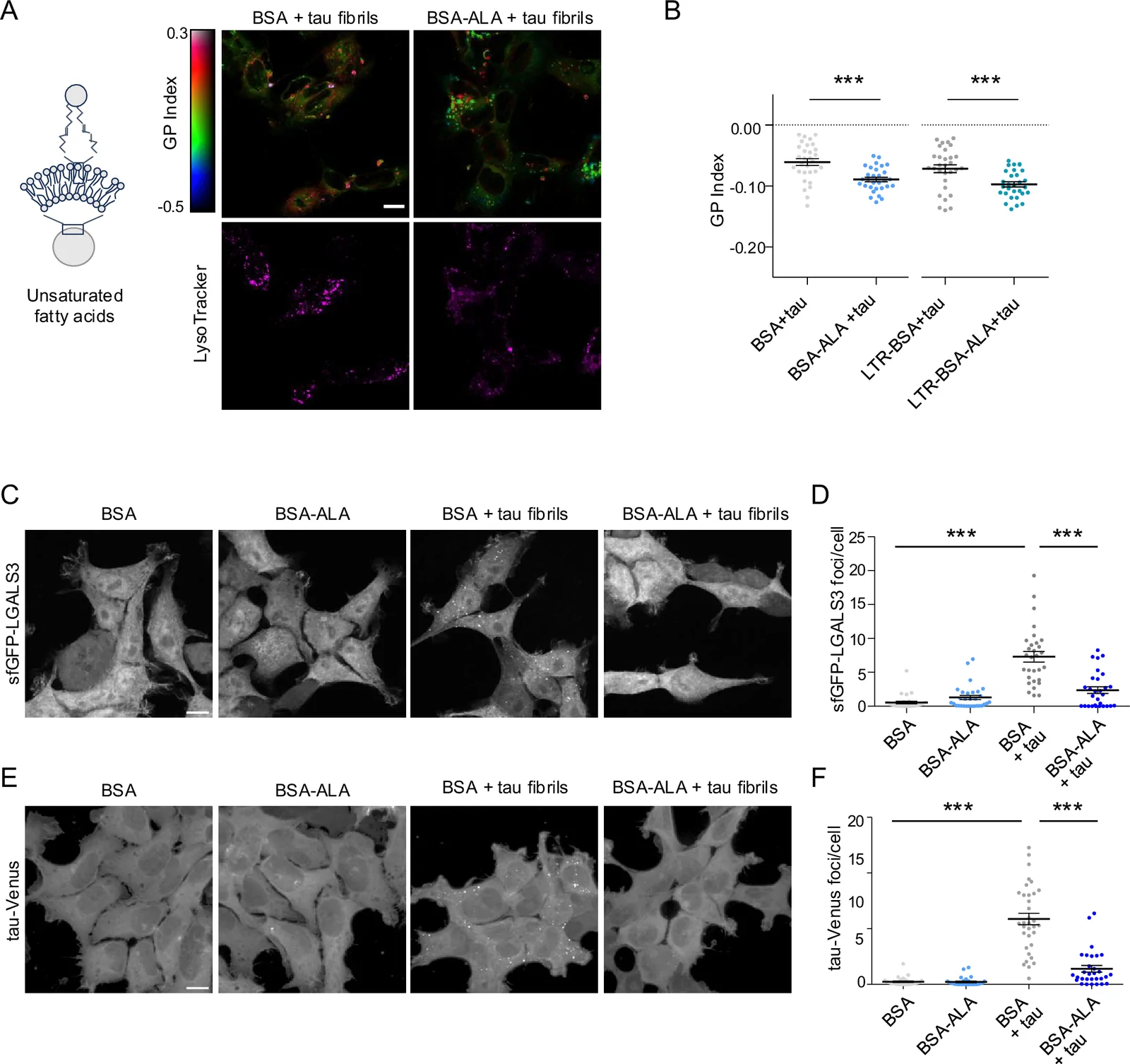
PUFA supplementation restores lysosomal membrane integrity and reduces seeded tau aggregation. (**A**) Left: unsaturated fatty acid membrane scheme. Right: Pseudo-colored images of SH-SY5Y cells pre-loaded with BSA control or 150 µM ALA conjugated to BSA (BSA–ALA) and treated with 1N4R tau fibrils showing the C-Laurdan GP Index at each pixel position. Lower panels show LysoTracker staining. Scale bar = 10 µm. (**B**) Quantification of GP values in SH-SY5Y cells pre-loaded with BSA control or 150 µM ALA conjugated to BSA (BSA–ALA) and exposed to 1N4R tau fibrils. GP values were measured across the whole cell (left) or restricted to LysoTracker-positive (LTR) regions (right). Statistical analysis was conducted using a two-way mixed-model ANOVA, followed by pairwise comparisons of estimated marginal means with Sidak correction for multiple comparisons. *n* = 3 independent experiments, with 10 images analyzed per experiment. (**C**) Maximum intensity projection of confocal z-stacks of HEK293T cells expressing sfGFP-LGALS3 pre-loaded with BSA or BSA–ALA with or without exposure to 1N4R tau fibrils. Scale bar = 10 µm. (**D**) Quantification of sfGFP-LGALS3 foci following indicated treatments. Statistical analysis comparing BSA + tau to other conditions was done using Kruskal–Wallis with Dunn’s post hoc test. *n* = 3 independent experiments, with 10 images analyzed per experiment. ***p < 0.001. (**E**) Maximum intensity projection of confocal z-stacks of tau-Venus biosensor cell line pre-loaded with BSA or BSA–ALA with or without exposure to 1N4R tau fibrils. Scale bar = 10 µm. (**F**) Quantification of tau-Venus foci following indicated treatments. Statistical analysis comparing BSA + tau to other conditions was done using Kruskal–Wallis with Dunn’s post hoc test. *n* = 3 independent experiments, with 10 images analyzed per experiment. ***p < 0.001. Figure 5—source data 1.This Source Data contains raw data for Figure 5.

We next asked whether increasing membrane fluidity could counteract these effects. Treatment of SH-SY5Y and HEK293T cells with the ω-3 PUFA α-linolenic acid (ALA) increased membrane fluidity (Figure 5—figure supplement 2A–C) and prevented tau fibril-induced membrane rigidification (Figure 5A, B, Figure 5—figure supplement 2D). Consistent with this, LysoTracker-based analysis indicated that ALA also affected lysosome-associated membrane properties. Moreover, ALA pre-treatment reduced tau fibril-induced endolysosomal rupture (Figure 5C, D, Figure 5—figure supplement 2E).

**Figure 5—figure supplement 1.**
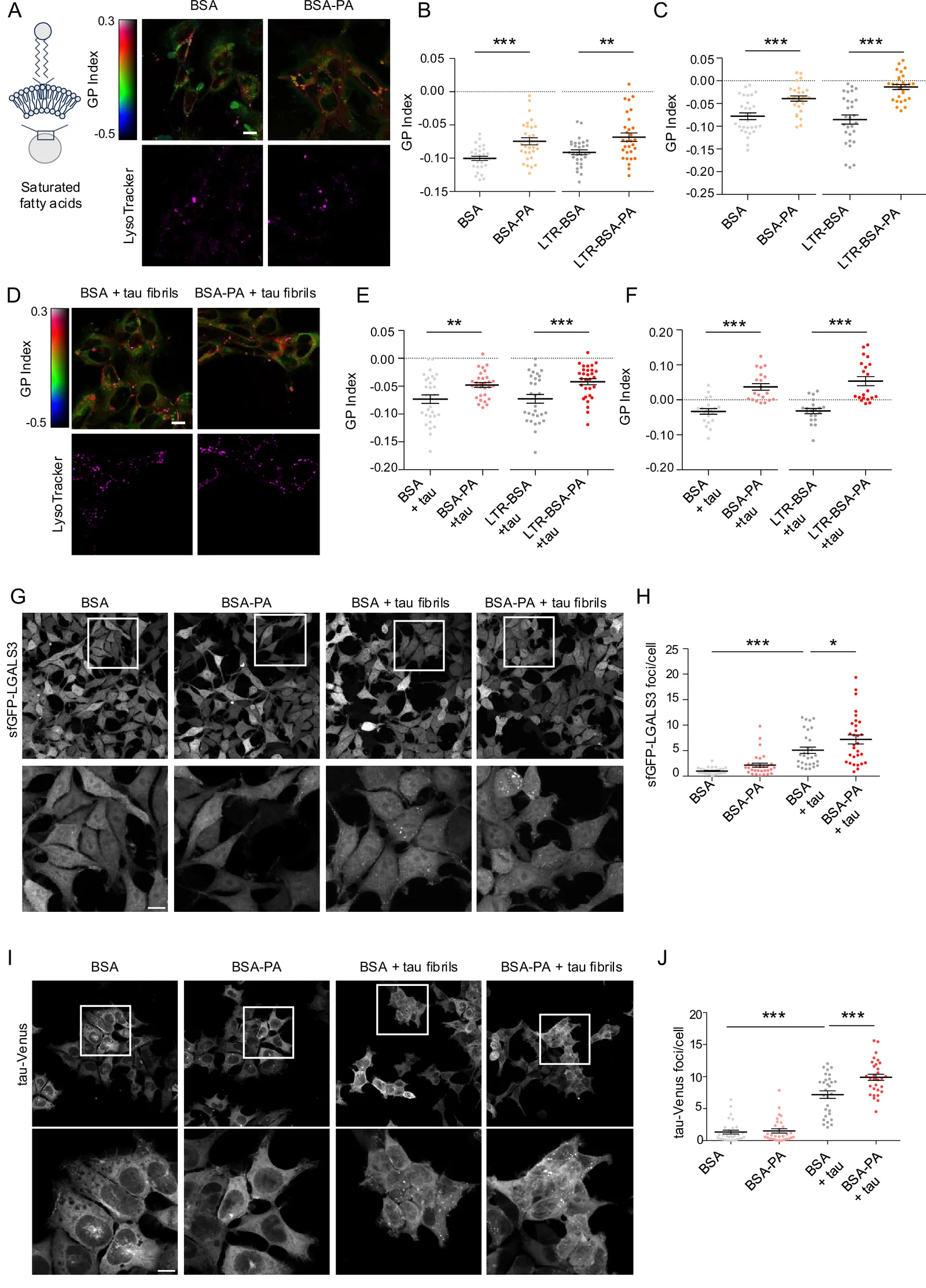
Saturated fatty acids decrease membrane fluidity and exacerbate seeded tau aggregation. (**A**) Left: saturated fatty acid membrane scheme. Right: upper panels: pseudo-colored images of SH-SY5Y cells pre-loaded with BSA or 50 µM PA conjugated to BSA (BSA–PA) displaying the C-Laurdan GP Index. Lower panels: LysoTracker staining. Scale bar = 10 µm. (**B**) Quantification of GP values in SH-SY5Y cells upon PA treatment. GP values were measured either across the whole cell (left) or specifically within LysoTracker-positive (LTR) regions (right). Statistical analysis was conducted using a two-way mixed-model ANOVA followed by pairwise comparisons of estimated marginal means with Sidak correction for multiple comparisons. *n* = 3 independent experiments, with 10 images analyzed per experiment. (**C**) Quantification of GP values in HEK293T cells after PA treatment. GP values were measured either across the whole cell (left) or specifically within LysoTracker-positive (LTR) regions (right). Statistical analysis was performed using a two-way mixed-model ANOVA, followed by pairwise comparisons of estimated marginal means with Sidak correction for multiple comparisons. *n* = 3 independent experiments, with 10 images analyzed per experiment. (**D**) Upper panels: Pseudo-colored images of SH-SY5Y cells pre-loaded with BSA or 50 µM PA conjugated to BSA (BSA–PA) and subsequently exposed to 1N4R tau fibrils displaying the C-Laurdan GP Index. Lower panels: LysoTracker staining. Scale bar = 10 µm. (**E**) Quantification of GP values in SH-SY5Y cells pre-loaded with PA and subsequently exposed to 1N4R tau fibrils. GP values were measured either across the whole cell (left) or specifically within LysoTracker-positive (LTR) regions (right). Statistical analysis was conducted using a two-way mixed-model ANOVA, followed by pairwise comparisons of estimated marginal means with Sidak correction for multiple comparisons. *n* = 3 independent experiments, with 10 images analyzed per experiment. (**F**) Quantification of GP values in HEK293T cells preloaded with PA and subsequently exposed to 1N4R tau fibrils. GP values were measured either across the whole cell (left) or specifically within LysoTracker-positive (LTR) regions (right). Statistical analysis was performed using a two-way mixed-model ANOVA, followed by pairwise comparisons of estimated marginal means with Sidak correction for multiple comparisons. *n* = 2 independent experiments, with 10 images analyzed per experiment. (**G**) Max. intensity projection of confocal z-stacks of HEK293T cells expressing sfGFP-LGALS3 pre-loaded with BSA or BSA–PA and subsequently exposed to PBS control or 1N4R tau fibrils. White box indicates the zoomed in section depicted in lower panel. Scale bar = 10 µm. (**H**) Quantification of sfGFP-LGALS3 foci in HEK293T cells pre-loaded with BSA or BSA–PA and subsequently exposed to PBS control or 1N4R tau fibrils. Statistical analysis comparing BSA + tau to other conditions was done using one-way ANOVA with Dunnett’s post hoc test. *n* = 3 independent experiments, with 10 images analyzed per experiment. (**I**) Max. intensity projection of confocal z-stacks of tau biosensor cells expressing Venus-tagged full-length P301S tau pre-loaded with BSA or BSA–PA with or without exposure to 1N4R tau fibrils. White box indicates the zoomed in section depicted in lower panel. Scale bar = 10 µm. (**J**) Quantification of P301S tau-Venus foci following indicated treatments. Statistical analysis comparing BSA + tau to other conditions was done using one-way ANOVA with Dunnett’s post hoc test. *n* = 3 independent experiments, 10 images analyzed per experiment. *p < 0.05, **p < 0.01, ***p < 0.001. Figure 5—figure supplement 1—source data 1.This Source Data contains raw data for Figure 5—figure supplement 1.

**Figure 5—figure supplement 2.**
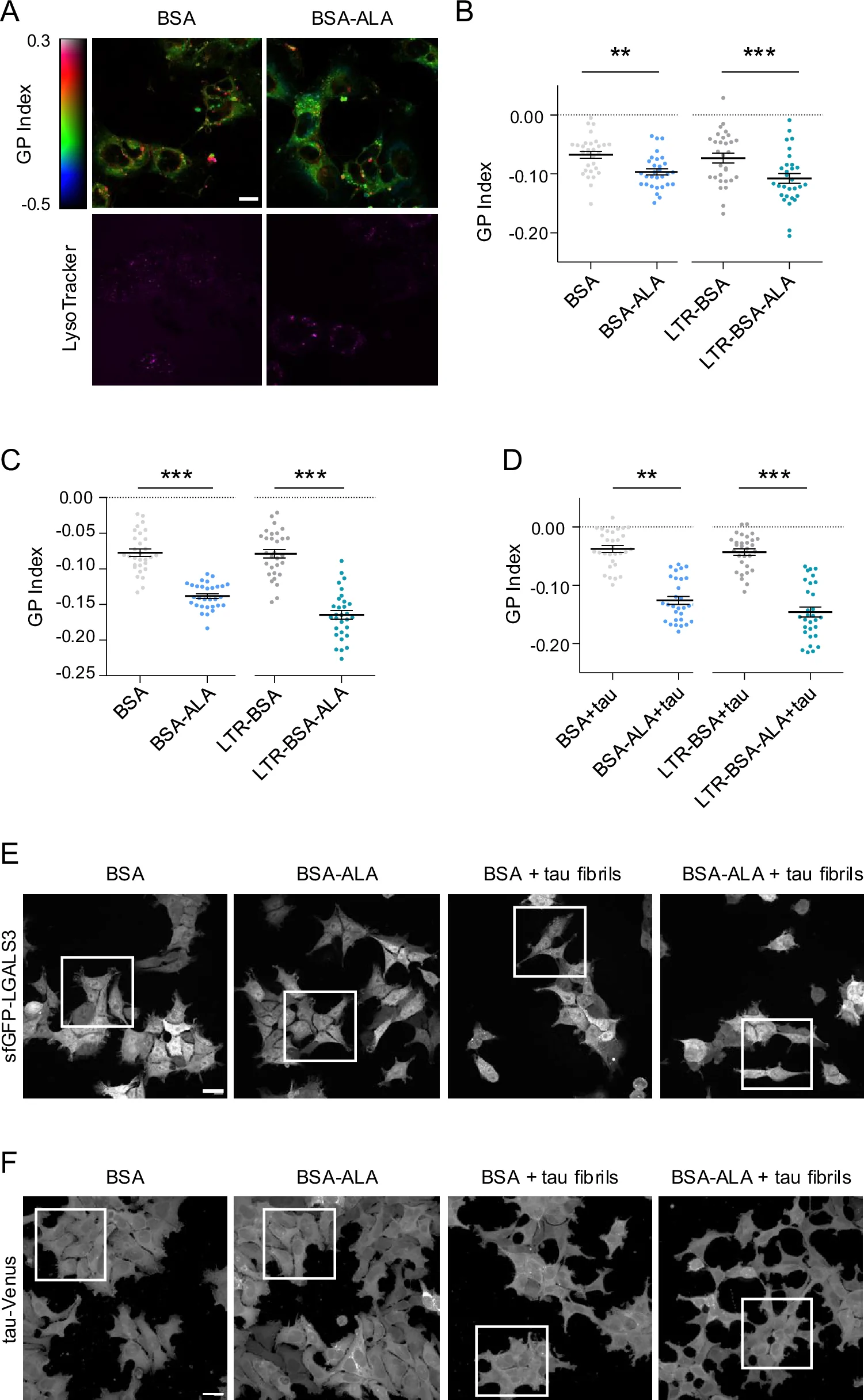
PUFA supplementation restores lysosomal membrane integrity and reduces seeded tau aggregation. (**A**) Upper panels: Pseudo-colored images of SH-SY5Y cells pre-loaded with BSA control or 150 µM ALA conjugated to BSA (BSA–ALA) displaying the GP Index. Lower panels: LysoTracker staining. Scale bar = 10 µm. (**B**) Quantification of GP values in SH-SY5Y cells pre-loaded with BSA or BSA–ALA. GP values were measured either across the whole cell or specifically within LysoTracker-positive (LTR) regions. Statistical analysis was conducted using a two-way mixed-model ANOVA with each experiment/image as random effects, with pairwise comparisons of estimated marginal means with Sidak correction for multiple comparisons. *n* = 3 independent experiments, 10 images each. Quantification of GP values in HEK293T cells pre-loaded with BSA or BSA–ALA and subsequently exposed to PBS control (**C**) or 1N4R tau fibrils (**D**). GP values were measured either across the whole cell or specifically within LysoTracker-positive (LTR) regions. Statistical analysis was conducted using a two-way mixed-model ANOVA followed by pairwise comparisons of estimated marginal means with Sidak correction for multiple comparisons. *n* = 3 independent experiments, with 10 images analyzed per experiment. (**E**) Max intensity projection of confocal z-stacks of HEK293T cells expressing sfGFP-LGALS3 pre-loaded with BSA or BSA–ALA with or without exposure to 1N4R tau fibrils. White box indicates the zoomed in section depicted in Figure 5C. Scale bar = 20 µm. (**F**) Max intensity projection of whole confocal z-stacks of P301S tau-Venus biosensor cell line pre-loaded with BSA or BSA–ALA with or without exposure to 1N4R tau fibrils. The white boxes delineate the zoomed images presented in Figure 5E. Scale bar = 20 µm. **p < 0.01, ***p < 0.001. Figure 5—figure supplement 2—source data 1.This Source Data contains raw data for Figure 5—figure supplement 2.

Since ALA prevented endolysosomal rupture, we expected it to also prevent seeded tau aggregation. Indeed, tau-Venus cells pre-loaded with ALA exhibited significantly fewer foci when exposed to tau fibrils (Figure 5E, F, Figure 5—figure supplement 2F). Together, these data show that increasing lysosomal membrane fluidity counteracts tau fibril-induced endolysosomal membrane damage and limits downstream seeded tau aggregation.

**Figure 6.**
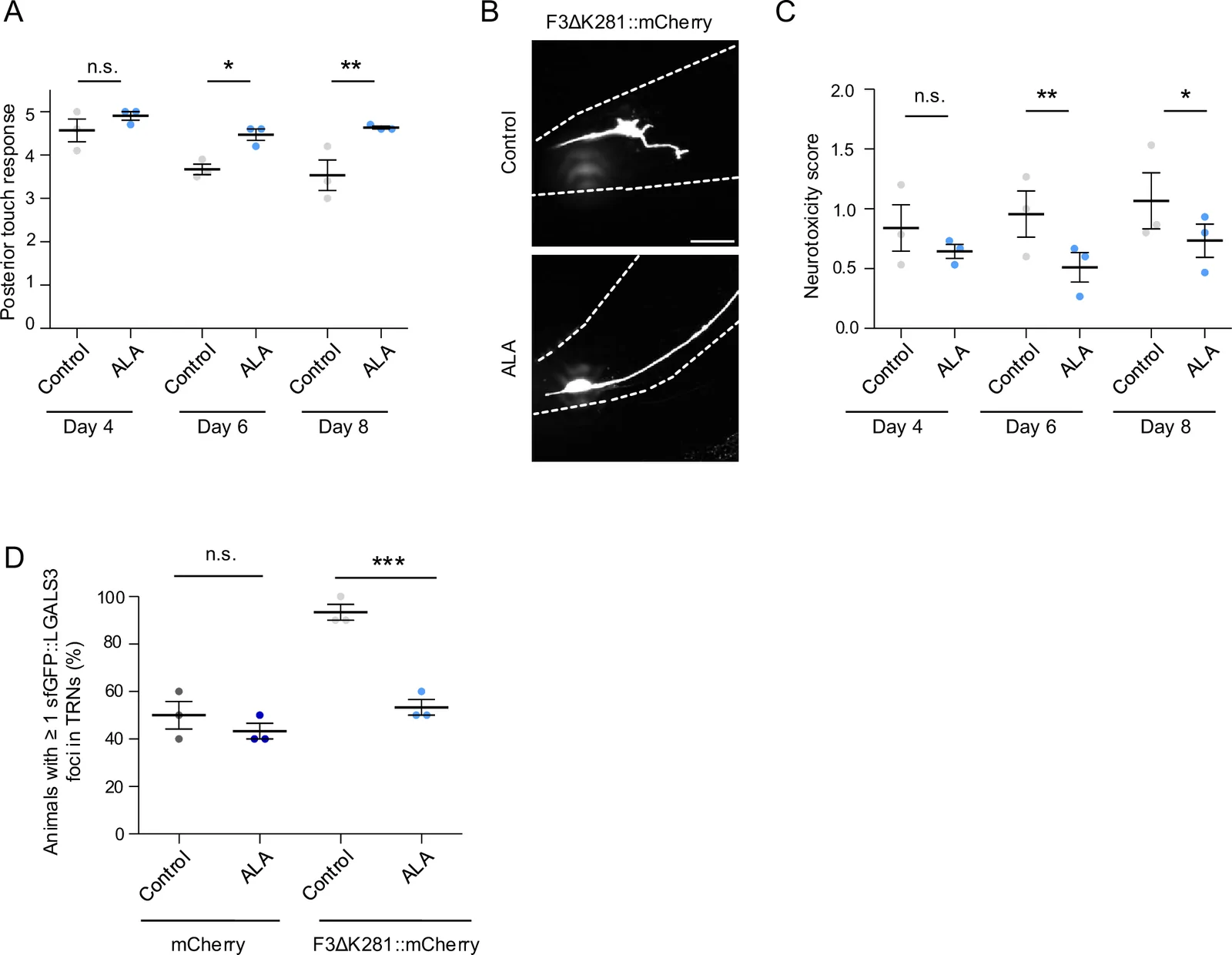
ALA improves neuronal function and reduces toxicity during aging. (**A**) Posterior touch response of animals expressing F3ΔK281::mCherry at indicated ages when grown on plates supplemented with ALA or ethanol solvent only control. Statistical analysis was done using two-way ANOVA with Bonferroni’s multiple comparison test. *n* = 3 independent experiments, with 10 animals analyzed per experiment. (**B**) Maximum intensity projection of confocal z-stacks of day 6 old animals expressing F3ΔK281::mCherry grown on EtOH solvent control or ALA plates. Scale bar = 20 µm. (**C**) Neurotoxicity score of PLM neurons of animals expressing F3ΔK281::mCherry grown on EtOH solvent control or ALA plates at indicated ages. Data were analyzed using a repeated-measures model of rank-transformed neurotoxicity scores with Type III Wald *χ*^2^ tests, followed by Bonferroni-adjusted pairwise comparisons of estimated marginal means. *n* = 3 independent experiments, with 15 animals analyzed per experiment. (**D**) Quantification of the percentage of animals with ≥1 sfGFP::LGALS3 puncta in touch receptor neurons, upon co-expression of either mCherry control or F3ΔK281::mCherry and growth on EtOH solvent control or ALA-supplemented plates on day 5 (second day of adulthood). Statistical analysis was done using two-way ANOVA with Bonferroni’s multiple comparison test. Each dot represents an independent experiment, lines indicate the mean ± SEM. *n* = 3 independent experiments, with 10 animals analyzed per experiment. n.s.: not significant, *p < 0.05, **p < 0.01, ***p < 0.001. Figure 6—source data 1.This Source Data contains raw data for Figure 6.

Finally, we asked whether ALA would also reduce the toxicity associated with aggregated tau in our *C. elegans* model. Expression of F3ΔK281::mCherry in touch receptor neurons resulted in an age-dependent decline in the response to gentle touch from day 4 (first day of adulthood) to day 8 (day 5 of adulthood) (Figure 6A, Figure 6—figure supplement 1; Sandhof et al., 2025). Supplementation with ALA significantly mitigated this behavioral deficit (Figure 6A, Figure 6—figure supplement 1) and reduced neurotoxicity (Figure 6B, C). Consistent with improved neuronal integrity, ALA treatment also reduced sfGFP::LGALS3 foci in touch receptor neurons (Figure 6D), indicating decreased endolysosomal damage in vivo.

**Figure 6—figure supplement 1.**
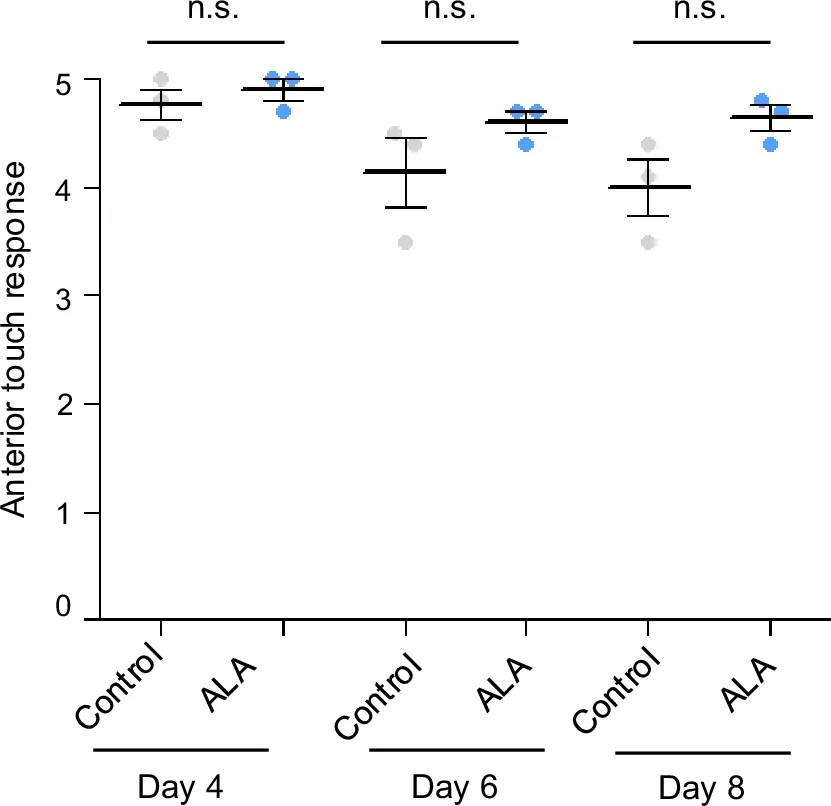
The impact of ALA on anterior touch response. Anterior touch response of animals expressing F3ΔK281::mCherry at indicated ages when grown on plates supplemented with ALA or ethanol solvent only control. Statistical analysis was done using two-way ANOVA with Bonferroni’s multiple comparison test. Two-way ANOVA showed significant effects of treatment (p = 0.0236) and age (p = 0.0391), but no treatment-by-age interaction (p = 0.4485). Bonferroni-adjusted comparisons between control and ALA at the individual ages were not significant. *n* = 3 independent experiments, with 10 animals analyzed per replicate. n.s.: not significant. Figure 6—figure supplement 1—source data 1.This Source Data contains raw data for Figure 6—figure supplement 1.

Together, these findings show that ALA-mediated increase in membrane fluidity reduces tau fibril-induced lysosomal membrane rigidification, endolysosomal damage, and seeded tau aggregation in human cells, while attenuating tau-associated neuronal dysfunction and endolysosomal damage in *C. elegans*.

## Discussion

**Figure 7.**
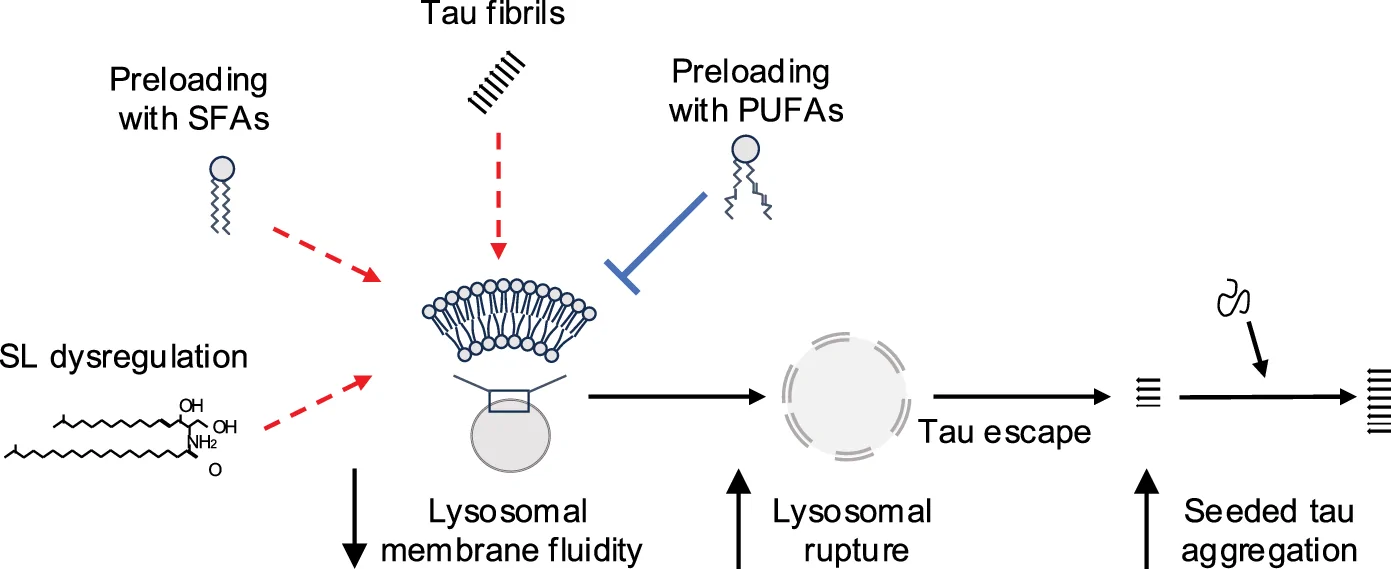
Proposed model linking membrane fluidity to seeded tau aggregation. Sphingolipid dysregulation or supplementation with saturated fatty acids reduces lysosomal membrane fluidity, promoting membrane rupture, tau seed escape, and seeded tau aggregation. In contrast, supplementation with polyunsaturated fatty acids increases membrane fluidity and protects lysosomal integrity.

Building on our previously published genome-wide screen in *C. elegans*, which identified SL metabolism as a regulator of endolysosomal integrity (Sandhof et al., 2025), we investigated how genetic perturbation of enzymes involved in SL metabolism promotes endolysosomal membrane rupture and tau-associated phenotypes. We found that disruption of SL metabolism decreases endolysosomal membrane fluidity, making vesicles more prone to rupture (Figure 7). Fibrillar tau had a similar effect on membrane rigidity and acted additively with perturbation of SL metabolism to exacerbate endolysosomal damage. In human cell models, disruption of SL metabolism enhanced tau-fibril-induced rupture and seeded tau aggregation, whereas increasing membrane fluidity with ALA reduced tau-induced membrane rigidification, endolysosomal damage, and seeded aggregation (Figure 7). In *C. elegans*, ALA attenuated aggregated tau-associated neuronal dysfunction and endolysosomal damage. Together, these findings support a model in which disruption of SL metabolism and tau accumulation converge on endolysosomal membrane rigidification, thereby promoting endomembrane rupture, and allowing tau seeds to escape into the cytosol, where they seed the aggregation of soluble endogenously expressed tau.

Our results show that reducing the expression of genes involved in both, biosynthesis and degradation of SLs significantly reduced the fluidity of endolysosomal membranes, making them more fragile and prone to rupture. KD of the saposin *spp-10*, the sphingosine kinase *sphk-1*, and the sphingosine-1-phosphate lyase *spl-1* blocks the degradation of complex SLs and should lead to an increase in their relative abundance, and therefore to a decrease in membrane fluidity. KD of the very-long-chain (3*R*)-3-hydroxyacyl-CoA dehydratase *hpo-8*, which is involved in fatty acid elongation, has been demonstrated to promote the incorporation of long-chain UFAs into phospholipids, thereby restoring plasma membrane fluidity (Ruiz et al., 2023). The absence of *hpo-8* leads to membrane rigidification (Ruiz et al., 2023). Conversely, KD of the serine incorporator *R11H6*.2 and the 3-ketodihydrosphingosine reductase *Y37E11AM.3*, which function in the SL de novo synthesis pathway, should theoretically decrease total SL content and consequently increase membrane fluidity. The cytochrome b5 reductases *hpo-19* and *T05H4.4*, required for lipid desaturation, are involved in the conversion of dihydroceramide to ceramide and the biosynthesis of PUFAs (Zhang et al., 2016); KD of these genes is expected to reduce the levels of SLs and PUFAs, which could theoretically either increase or decrease membrane fluidity, depending on which metabolic pathway is predominantly affected.

Our observation that KD of all these genes results in decreased membrane fluidity is therefore seemingly counterintuitive. However, SLs form a complex metabolic network and can interconvert, allowing dynamic adaptation of SL levels. In addition, cells may compensate for the loss of certain SLs by altering the biosynthesis or turnover of other lipid molecules. This dynamic interplay makes it difficult to predict how individual gene knockdowns affect endolysosomal membrane composition and biophysical properties. Thus, genes that act at apparently opposing steps of SL metabolism could still converge on similar changes in membrane packing and fluidity.

Lipidomic analyses will be important to determine how SL perturbations remodel cellular and organellar lipid composition. However, even detailed lipidomics would not by itself identify which lipid changes are responsible for the observed membrane rigidification. Indeed, membrane fluidity is not determined by a single lipid species, but by the combined properties of the entire membrane, including lipid abundance, saturation, acyl-chain length, head groups, sterol content, and membrane-associated proteins. Thus, an increase or decrease in a given lipid species cannot be directly translated into a predictable change in membrane fluidity without additional functional validation. Future lysosome-enriched or organelle-specific lipidomic approaches, ideally complemented by analyses of membrane-associated proteins, will therefore need to be combined with direct manipulation of candidate lipid or protein species, followed by measurements of membrane fluidity and rupture, to determine which molecular changes causally contribute to endolysosomal membrane rigidification.

Importantly, endolysosomal membrane rupture and global lysosomal function are related but not identical readouts. This distinction is supported by work showing that lipid dysregulation can induce lysosomal membrane permeabilization and lysosomal accumulation of endogenous protein aggregates without broadly impairing core lysosomal or proteasomal functions (Yong et al., 2024). Thus, membrane damage can occur even when general lysosomal activity is not overtly disrupted. Conversely, a recent study independently identified SPHK-1 as an important regulator of lysosomal integrity in *C. elegans*, showing that strong *sphk-1* loss-of-function causes lysosomal sphingosine accumulation, membrane rupture, impaired degradative function, cargo accumulation, developmental defects, and reduced lifespan (Li et al., 2026a). Together, these studies suggest that the functional consequences of disrupted SL metabolism can vary depending on allele strength, tissue, developmental stage, and assay conditions. In the present study, we focused on endolysosomal membrane fluidity and rupture because these membrane-level changes are directly linked to tau seed escape and seeded aggregation.

The central nervous system is particularly rich in lipids. Approximately 50% of the brain’s dry weight consists of lipids with high concentrations of cholesterol, phospholipids, and sphingolipids (Yoon et al., 2022; Hornemann, 2021). Alterations in lipid and SL metabolism have been observed not only during the aging process, but also in various late-onset neurodegenerative diseases including AD (van Echten-Deckert and Walter, 2012; Uranbileg et al., 2024). While aging is the major risk factor for AD, one of the most significant genetic risk factors for AD is the apolipoprotein E (ApoE) polymorphism, specifically the ApoE4 allele (Corder et al., 1993). ApoE is the primary apolipoprotein in the brain, crucial for lipid transport and metabolism. Besides cholesterol, the ApoE4 variant has been shown to influence SL metabolism, thereby affecting the pathogenesis of AD from its early stages (van Echten-Deckert and Walter, 2012; Uranbileg et al., 2024; Kosicek et al., 2012). Thus, although disturbances in SL homeostasis are increasingly recognized in AD, the mechanisms by which they contribute to disease progression remain poorly understood.

AD brains exhibit elevated levels of sphingosine, ceramide 1-phosphate, and ceramide compared to control subjects (Uranbileg et al., 2024). Multivariate analysis and machine learning also identified these SL species as key contributors to AD (Uranbileg et al., 2024). However, these analyses were performed in different brain regions without cell type or subcellular resolution. We found that perturbation of SL metabolism primarily affected endolysosomal membranes, with little effect on plasma membrane fluidity in *C. elegans*. In human cells, LysoTracker-based analysis further revealed pronounced increases in GP values within lysosome-associated compartments. The lysosomal membrane is particularly rich in sphingolipids (Fabri et al., 2020), which may make it especially sensitive to perturbation of SL metabolism. Studies in yeast have shown that increased membrane lipid saturation affects the vacuole – the functional counterpart of mammalian lysosomes – as well as the nuclear envelope, whereas the plasma membrane and mitochondria remained relatively unaffected (Romanauska and Köhler, 2023). These findings indicate that cellular membranes vary in their resilience to lipid saturation stress, potentially due to differences in lipid composition and protective mechanisms (Romanauska and Köhler, 2023; Harayama and Riezman, 2018). To better understand the implications of SL alterations in AD, it would be important to examine whether the observed changes primarily originate from endolysosomal membranes or also from other organelles, such as the endoplasmic reticulum and the nucleus, or from the plasma membrane.

We observed that fibrillar tau also increased membrane rigidity and exacerbated endolysosomal rupture. This suggests that tau accumulation and disruption of SL metabolism may converge on a common biophysical mechanism. This interpretation is consistent with recent ultrastructural studies showing that intralysosomal amyloid assemblies can physically deform and rupture lysosomal membranes (Elias et al., 2025; Li et al., 2026b). Whether this mechanism is specific to tau or also applies to other amyloid assemblies remains to be determined. In *C. elegans*, tau transmission sensitized endolysosomal membranes to SL perturbation, especially under sub-saturating RNAi conditions. The enhanced rupture phenotype is unlikely to result from a direct effect of RNAi on neuronal F3ΔK281::mCherry expression, as *C. elegans* neurons are largely refractory to systemic RNAi under the conditions used here (Calixto et al., 2010). Consistent with this, *sphk-1* RNAi did not alter hypodermal F3ΔK281::mCherry levels, supporting the interpretation that SL perturbation increases endolysosomal membrane damage rather than tau transmission itself.

These findings also raise the possibility that alterations in SL metabolism are not only a cause but also a consequence of tau-associated pathology (van Echten-Deckert and Walter, 2012; Uranbileg et al., 2024). Accumulation of misfolded tau in lysosomes could impair lysosomal clearance capacity and alter lipid turnover, leading to accumulation of membrane lipids or other biomolecules. In turn, impaired SL metabolism could further reduce endolysosomal membrane fluidity and promote rupture. Such a cycle, in which imbalances in proteostasis and lipostasis negatively influence each other, may contribute to progressive lysosomal dysfunction and neurodegeneration (Tittelmeier and Nussbaum-Krammer, 2025).

Manipulating membrane fluidity independently of SL enzymes further supported a role for membrane biophysics in tau-induced endolysosomal damage. Increasing membrane rigidity with PA exacerbated tau-induced rupture and seeded aggregation, whereas increasing membrane fluidity with ALA reduced tau-induced lysosomal membrane rigidification, endolysosomal damage, and seeded aggregation in cells and attenuated tau-associated neuronal dysfunction in *C. elegans*.

Given the high levels of saturated and trans fats in the Western diet (Rakhra et al., 2020), it is plausible that dietary factors contribute to the dysregulation of lipid homeostasis in the brain. In line with a broader role of lipid saturation in AD, a recent plasma lipidomics study reported reduced highly unsaturated lipid species and increased saturated lipid species in women with AD (Wretlind et al., 2025). The Mediterranean UFA-rich diet has been associated with healthy aging, while reduced UFA brain levels have been correlated with increased tau and Aβ pathology and decreased cognitive function in AD (Snowden et al., 2017; Bischoff-Ferrari et al., 2025). Our findings therefore provide a possible mechanistic explanation for the beneficial effects of PUFA-rich diets or PUFA supplementation reported in AD-related contexts (Shinto et al., 2014; Wood et al., 2022). However, PUFAs can also influence lipid signaling, oxidative stress responses, and broader membrane remodeling. We therefore cannot exclude additional direct or indirect effects of ALA. Nevertheless, the opposing effects of PA and ALA, together with the SL knockdown data, support the interpretation that membrane fluidity is a major determinant of endolysosomal membrane integrity and rupture in our models.

In sum, our study identifies endolysosomal membrane fluidity as a key determinant of vesicle integrity in the context of disrupted SL metabolism and tau accumulation. We propose that perturbation of SL metabolism and fibrillar tau converge on membrane rigidification, thereby reducing endolysosomal membrane integrity and promoting vesicle rupture, tau seed escape, and seeded tau aggregation. Restoring membrane fluidity may therefore represent a strategy to limit tau-induced endolysosomal damage and tau-associated toxicity, although the lipid species, membrane compartments, and additional cellular pathways contributing to this protection remain to be defined.

## Materials and methods

**Key resources table:** 

Reagent type (species) or resource	Designation	Source or reference	Identifiers	Additional information
Strain (*Caenorhabditis elegans*)	See Supplementary file 1 for details.	See Supplementary file 1 for details.	See Supplementary file 1 for details.	See Supplementary file 1 for details.
Strain (*Escherichia coli* OP50 and HT115 RNAi clones)	See Supplementary file 2 for details.	See Supplementary file 2 for details.	See Supplementary file 2 for details.	See Supplementary file 2 for details.
Cell line (*Homo sapiens*)	SH-SY5Y	ATCC	ATCC:CRL-2266 RRID:CVCL_0019	Human neuroblastoma cell line.
Cell line (*Homo sapiens*)	HEK293T	ATCC	ATCC:CRL-3216 RRID:CVCL_0063	Human embryonic kidney-derived cell line.
Cell line *(Homo sapiens*)	HEK293T sfGFP-LGALS3	Sandhof et al., 2020; DOI:10.1080/15548627.2019.1643657		Stable HEK293T reporter cell line expressing sfGFP-LGALS3.
Cell line (*Homo sapiens*)	HEK293T 0N4R tauP301S-Venus (tau-Venus biosensor)	Dr. William A. McEwan, Cambridge University; published (McEwan et al., 2017; Nachman et al., 2020)		HEK293T biosensor line expressing Venus-tagged full-length P301S mutant 0N4R tau.
Recombinant DNA reagent	rgef-1p::sfGFP::LGALS3::tbb2-3′UTR expression plasmid	This paper		pCFJ150-based vector backbone with rgef-1 promoter, sfGFP::LGALS3, and tbb-2 3′UTR.
Recombinant DNA reagent	pPD49.26-sfGFP::LGALS3	Dr. Bin Liu and Dr. Marja Jäättelä, University of Copenhagen		Expression plasmid coding for sfGFP::LGALS3, shared by Bin Liu and Marja Jäättelä.
Recombinant DNA reagent	pLenti PGK Puro DEST (w529-2)	Addgene; Campeau et al., 2009; DOI:10.1371/journal.pone.0006529	Addgene:19068 RRID:Addgene_19068	Gift from Dr. Eric Campeau and Dr. Paul Kaufman; ordered from Addgene.
Sequence-based reagent	ON-TARGETplus Human SPHK2 siRNA SMARTpool	Horizon Discovery (Dharmacon)	Horizon Discovery:M-004831-00-0005	Used at 20 nM final concentration for SPHK2 knockdown in human cells.
Sequence-based reagent	ON-TARGETplus non-targeting control siRNA pool	Horizon Discovery (Dharmacon)		Used as control for siRNA-mediated knockdown experiments.
Peptide, recombinant protein	Full-length human 1N4R tau monomer	Prepared as described in Tardivel et al., 2016; DOI:10.1186/s40478-016-0386-4		Purified recombinant human Tau-412 (1N4R) used for monomeric tau controls and fibril assembly.
Peptide, recombinant protein	Full-length human 1N4R tau fibrils	This paper; prepared from purified recombinant 1N4R tau		Assembled at 40 μM with 10 μM heparin for 5 days at 37°C; fragmented by sonication; used at 400 nM for seeding and membrane-fluidity experiments.
Antibody	Rabbit polyclonal anti-SPHK2	Proteintech (Thermo Fisher/Life technology)	17096-1-AP RRID:AB_10598479	Immunoblotting primary antibody; 1:5000.
Antibody	Mouse monoclonal anti-GAPDH (clone GAPDH-71.1)	Sigma-Aldrich/Merck	Sigma-Aldrich:G8795; clone:GAPDH-71.1 RRID:AB_1078991	Immunoblotting loading-control primary antibody; 1:5000.
Antibody	Goat anti-mouse IgG (H+L), HRP conjugate	Bio-Rad	Bio-Rad:1706516 RRID:AB_2921252	Secondary antibody for ECL-based immunoblot detection.
Antibody	Goat anti-rabbit IgG (H+L), HRP conjugate	Bio-Rad	Bio-Rad:1706515 RRID:AB_11125142	Secondary antibody for ECL-based immunoblot detection.
Commercial assay or kit	Mycoplasma testing	GATC Biotech		Cell lines were regularly tested for Mycoplasma contamination.
Commercial assay or kit	Protein assay dye reagent concentrate	Bio-Rad	Bio-Rad:5000006	Used to determine protein concentration before SDS-PAGE/immunoblotting.
Chemical compound, drug	C-Laurdan	Tocris Bioscience/Bio-Techne	Tocris:7273; CAS:959839-06-6	Membrane-order/fluidity dye; used at 10 mM for live worms and 15 μM for cells.
Chemical compound, drug	LysoTracker Red DND-99	Invitrogen/Thermo Fisher Scientific	Invitrogen:L7528	Used at 50 nM with C-Laurdan to identify LysoTracker-positive regions in cells.
Chemical compound, drug	α-Linolenic acid (ALA)	Sigma-Aldrich/Merck	Sigma-Aldrich:L2376; CAS:463-40-1	Omega-3 polyunsaturated fatty acid; conjugated to fatty-acid-free BSA for cells and supplemented in *C. elegans* NGM at 0.3 mM.
Chemical compound, drug	Palmitic acid (PA)	Sigma-Aldrich/Merck	Sigma-Aldrich:P0500; CAS:57-10-3	Saturated fatty acid; conjugated to fatty-acid-free BSA for cells or used to enrich OP50 bacterial food source for *C. elegans* experiments.
Chemical compound, drug	Fatty-acid-free BSA	Sigma-Aldrich/Merck	Sigma-Aldrich:A8806; CAS:9048-46-8	Used to conjugate ALA and PA for cell treatments.
Chemical compound, drug	NP-40 substitute	Sigma-Aldrich/Merck	Sigma-Aldrich:74385; CAS:9016-45-9	Used at 0.001% (vol/vol) to improve fatty acid distribution in *C. elegans* NGM plates.
Chemical compound, drug	Lipofectamine 2000 Transfection Reagent	Thermo Fisher Scientific	Thermo Fisher Scientific:11668019	Transfection reagent used for siRNA delivery into human cells; medium exchanged after 6 hr.
Chemical compound, drug	ATTO 550 NHS ester	ATTO-TEC GmbH (Leica Microsystems)		Used to label 1N4R tau fibrils.
Chemical compound, drug	Levamisole hydrochloride	AppliChem	AppliChem:A4341; CAS:16595-80-5	Used at 2% (wt/vol) in mounting mix for live *C. elegans* imaging.
Chemical compound, drug	Nanosphere Size Standard, 100 nm	Thermo Scientific	Thermo Scientific: 3100A; Fisher Scientific: 10671531	Used at 50% (vol/vol) in mounting mix for live *C. elegans* imaging.
Chemical compound, drug	NheI restriction enzyme	New England Biolabs	NEB:R0131	Used for restriction digestion during cloning.
Chemical compound, drug	BglII restriction enzyme	New England Biolabs	NEB:R0144S	Used for restriction digestion during cloning.
Chemical compound, drug	Deoxynucleotide (dNTP) Solution Set	New England Biolabs	NEB:N0446S	Used to prepare the Gibson assembly reaction mix.
Chemical compound, drug	β-Nicotinamide adenine dinucleotide (NAD+)	New England Biolabs	NEB:B9007S	Used in Gibson assembly reaction mix.
Chemical compound, drug	T5 Exonuclease	New England Biolabs	NEB:M0663S	Used in Gibson assembly reaction mix.
Chemical compound, drug	Phusion High-Fidelity DNA Polymerase	Thermo Fisher Scientific	Thermo Fisher Scientific:F530	Used in Gibson assembly reaction mix.
Chemical compound, drug	Taq DNA Ligase	New England Biolabs	NEB:M0208L	Used in Gibson assembly reaction mix.
Software, algorithm	FIJI/ImageJ	Schindelin et al., 2012; https://imagej.net/software/fiji/	RRID:SCR_002285; RRID:SCR_003070	Used for image processing, thresholding, particle analysis, and foci quantification.
Software, algorithm	Automated ImageJ macro for C-Laurdan GP analysis	Published guidelines cited as (Owen et al., 2011); this paper		Used to calculate GP values and extract LysoTracker-positive regions.
Software, algorithm	GraphPad Prism	GraphPad Software	Version 6h; version 10.1.1 RRID:SCR_002798	Used for statistical analyses and graphing.
Software, algorithm	R	R Foundation for Statistical Computing	Version 4.3.1 RRID:SCR_001905	Used for statistical analyses and graphing.
Software, algorithm	R package nlme	CRAN	Version 3.1-166	Used for statistical analyses and graphing.
Software, algorithm	R package lme4	CRAN	Version 1.1-35.5	Used for statistical analyses and graphing.
Software, algorithm	R package emmeans	CRAN	Version 1.10.6	Used for statistical analyses and graphing.
Software, algorithm	R package dplyr	CRAN	Version 1.1.4	Used for statistical analyses and graphing.
Software, algorithm	Leica acquisition software	Leica Microsystems		Used for C-Laurdan image acquisition on DMI6000 and Leica SP8X WLL microscopes.
Other	Olympus IXplore SpinSR confocal microscope	Olympus/Evident		Used for confocal imaging of galectin puncta and tau-Venus foci.
Other	Zeiss LSM 780 confocal microscope	Carl Zeiss		Used for FRAP measurements of *C. elegans* membranes.
Other	Leica M205 FA widefield binocular microscope	Leica Microsystems		Used for scoring hypodermal sfGFP::LGALS3 foci in *C. elegans*.
Other	Leica SP8X WLL microscope	Leica Microsystems		Used for C-Laurdan imaging.
Other	Hielscher Vial Tweeter/ultrasonic processor UIS250v	Hielscher Ultrasonics	UIS250v	Used to fragment 1N4R tau fibrils for seeding experiments.

### Maintenance of *C. elegans,* RNAi experiments, and age synchronization

*C. elegans* (hermaphrodites, no males) were cultured using standard methods (Brenner, 1974). If not otherwise indicated, worms were grown on nematode growth medium (NGM) plates seeded with *E. coli* strain OP50 at 20°C. For RNAi, NGM medium was supplemented with ampicillin, tetracycline, and IPTG, seeded with the respective HT115 *E. coli* RNAi clones (from the Ahringer RNAi library, for details see Supplementary file 2) and grown at 20°C. For diluted RNAi experiments, RNAi was diluted with EV control bacteria. Animals were age-synchronized by bleaching. Briefly, gravid adults were dissolved in 20% sodium hypochlorite solution. The surviving *C. elegans* embryos were hatched overnight in M9 buffer with gentle rocking at 20°C. The next day, appropriate amounts of L1 larvae were added to the plates.

### Cloning and generation of the neuronal sfGFP::LGALS3 strain

The sfGFP::LGALS3 reporter sequence was amplified from a previously cloned plasmid (Sandhof et al., 2025) via PCR using suitable primers for subsequent Gibson assembly. The pCFJ150 vector backbone already containing an rgef-1p::GFP1-10::tbb2-3′UTR construct was cut via restriction digestion using NheI (NEB #R0131) and BglII (NEB **#**R0144) to remove the GFP1-10 sequence. The sfGFP::LGALS3 construct was subsequently cloned into this vector backbone in a Gibson assembly reaction using 0.08 pmol of plasmid backbone and a fivefold molar excess of insert. 10 µl of fragment-vector mix was added to 10 µl Gibson assembly reaction mix containing 1.3x ISO buffer (6.5% wt/vol PEG-8000 [Sigma-Aldrich 89510], 130 mM Tris-HCl (Carl Roth, 4855.2) pH = 7.5, 13 mM MgCl_2_ (Carl Roth, A537.1), 13 mM DTT (Sigma-Aldrich 43816), 0.26 mM each dATP, dTTP, dCTP, and dGTP (NEB, N0446), 1.3 mM NAD (NEB, B9007S)), 0.05 U T5 exonuclease [NEB, M0663], 0.3 U Phusion polymerase (Thermo Fisher Scientific, F530), and 50 U Taq ligase [NEB, M0208L] and incubated at 50°C for 2 hr. Subsequently, the whole reaction was used to transform chemically competent *E. coli*. The extrachromosomal array was created by microinjecting the expression plasmid rgef-1p::sfGFP::LGALS3::tbb2-3′UTR (30 ng/µl) into the gonads of young adult wildtype *C. elegans* (N2s), together with a co-injection marker (15 ng/µl) for expression of RFP in coelomocytes. All strains generated or used in this study are found in Supplementary file 1.

### *C. elegans* fatty acid supplementation

PUFAs were dissolved in EtOH and stored in the dark at –20°C under a N_2_ atmosphere to prevent oxidation. NGM was cooled after autoclaving to 55°C. To enhance fatty acid distribution, NP-40 substitute (0.001% vol/vol) was added to the media and subsequently either 0.3 mM of the desired fatty acid solution or the ethanol solvent as control and stirred for 5 min before plate pouring (Svensk et al., 2013). PA was supplemented by enriching the OP50 bacterial food source (Ruiz et al., 2018). Overnight cultures were grown in the presence of 2 mM of PA dissolved in EtOH or EtOH alone. The next day, OP50 bacteria were pelleted by centrifugation (10 min at 4500 × *g*) and washed 2x with M9 buffer (Devkota et al., 2017). 10x concentrated bacteria (in M9) were then seeded on NGM plates.

### Quantification of lysosomal rupture (galectin puncta assay) in *C. elegans*

Quantification of animals showing hypodermal lysosomal rupture was done as previously described (Aits et al., 2015; Sandhof et al., 2020). Briefly, age-synchronized animals expressing the sfGFP::LGALS3 construct in the hypodermis were seeded on OP50 or RNAi NGM plates. Scoring was done on day 5 (second day of adulthood) using a Leica M205 FA widefield binocular microscope. 20–50 worms were analyzed per replicate. *Sphk-1* mutant animals were obtained from CGC (CZ24969; *sphk-1(ju831)*) and crossed with animals expressing sfGFP::LGALS3 in the hypodermis.

Formation of sfGFP::LGALS3 foci in touch receptor neurons was quantified in day 5 (second day of adulthood) old animals co-expressing either mCherry or F3ΔK281::mCherry in touch receptor neurons using an Olympus IXplore SpinSR confocal microscope equipped with a UPlanSApo 60×/1.30 silicone oil objective. Foci formation was checked using a 488-nm laser at 80% power with an exposure time of 200 ms. An animal was counted as positive for endolysosomal rupture if it had at least one sfGFP::LGALS3 focus in the PLM or ALM neurons.

### Mounting of live animals for imaging

Synchronized animals were mounted on 8–10% agarose in M9 buffer pads with a drop of mounting mix (2% (wt/vol) levamisole and 50% (vol/vol) nanosphere size standards (Thermo Fisher)) and covered with a coverslip.

### Fluorescence recovery after photobleaching

FRAP measurements to estimate membrane fluidity were done following a published protocol (Devkota and Pilon, 2018). Animals were synchronized by bleaching and grown until day 5 (second day of adulthood) on the indicated RNAi or empty vector (EV) control plates. Directly before the FRAP experiments, animals were mounted at the Zeiss LSM 780 confocal microscope. FRAP measurements of either GFP enriched in the intestinal plasma membrane, or LAAT-1::mCherry located in intestinal or hypodermal lysosomal membranes were taken with 40X Water immersion objective. For the lysosomal membrane, mCherry-positive membranes were photobleached over a circular area (seven pixel radius) using 10 iterations of the 561 nm DPSS laser with 100% laser power transmission. Images were collected at a 12-bit intensity resolution over 128 × 128 pixels (digital zoom 6X) using a pixel dwell time of 3.15 μsec with 2% laser power transmission. For the plasma membrane, GFP-positive membranes were photobleached over a circular area (10 pixel radius) using 20 iterations of the 488 nm Argon laser with 100% laser power transmission. Images were collected at a 12-bit intensity resolution over 128×128 pixels (digital zoom 6X) using a pixel dwell time of 3.15 μsec with 10% laser power transmission. At least 6 pre-bleaching images were collected before the region of interest was bleached. The recovery of fluorescence was traced for 25 s. Fluorescence recovery and *t*_half_ were calculated as previously described in Svensk et al., 2016.

### Quantification of spreading

Transmission of F3ΔK281::mCherry was quantified as previously published in Sandhof et al., 2025. In short, the hypodermis of age-synchronized worms expressing F3ΔK281::mCherry in touch receptor neurons was imaged on day 5 (second day of adulthood) using an Olympus IXplore SpinSR confocal microscope equipped with a UPlanSApo 60×/1.30 silicone oil objective. Images were acquired using a 561-nm laser at 30% power with an exposure time of 450 ms. Z-stacks were collected at 0.35 μm intervals, and hypodermal fluorescence intensity (integrated density) was quantified using ImageJ.

### Touch sensory assay and neurotoxicity in touch receptor neurons

Touch sensory assay was done as previously described (Chalfie et al., 1985). Briefly, touch sensitivity was tested by gently stroking the tip of an eyebrow hair attached to a toothpick transversely across the anterior or posterior half of an animal. A touch-sensitive animal was one that stopped/moved away from the stimulus. Ten worms were examined for each strain in three biological replicates with five strokes in the anterior and posterior. For neurotoxicity scoring of the PLM neurons, live animals were imaged using an Olympus IXplore SpinSR Confocal microscope with a UplanS Apo 60x/1.30 silicone oil objective. The neurotoxicity score was determined by adding a score of 1 for each of the following phenotypes: soma outgrowth, process branching, process break, wavy process. Fifteen animals were examined at each day of age in three independent biological replicates.

### Cell culture

This work involved the use of human cell lines, including SH-SY5Y neuroblastoma cells, HEK293T cells, HEK293T cells stably expressing sfGFP-LGALS3, and HEK293T tau biosensor cells expressing Venus-tagged full-length P301S mutant 0N4R tau (0N4R tauP301S-Venus). SH-SY5Y and HEK293T cells were obtained from ATCC. The HEK293T tau-Venus biosensor cell line was kindly provided by Dr. William A. McEwan, Cambridge University (McEwan et al., 2017). Cell line identity was not independently authenticated by STR profiling or another authentication method in the course of this study. All cell lines were routinely tested for mycoplasma contamination and tested negative. To our knowledge, none of the cell lines used is listed in the ICLAC Register of Misidentified Cell Lines.

Cells were cultured in DMEM containing high glucose, GlutaMAX Supplement, pyruvate (Gibco), and 10% FBS (Gibco) at 37°C and 5% CO_2_. Regular mycoplasma tests were performed and cells were tested negative (GATC Biotech).

### Quantification of lysosomal rupture (galectin puncta assay) and tau-Venus foci in cells

Cells were imaged on an Olympus IXplore SpinSR Confocal microscope with a UplanS Apo 60x/1.30 Silicon oil objective. To detect the number of foci in an image, a difference of Gaussian blur was applied to maximum projections to isolate foci from cytosolic sfGFP or Venus signal, respectively. Thresholding was applied using ‘moments’ threshold on FIJI. Number of foci was counted using the ‘analyze particles’ function. Cell number was counted manually to obtain number of foci/cell for each image.

### C-Laurdan dye measurement of membrane fluidity in worms and cells

Live worms were stained with 10 mM C-Laurdan dye (6-dodecanoyl-2-dimethylaminonaphthalene) (Tocris Bioscience/Bio-Techne) as previously described (Jeong et al., 2023). ROI was drawn around the soma of the PLM using the mCherry signal and only the GP values in this ROI were collected. Cells were also stained with C-Laurdan at 15 µM for 2 hr in combination with LysoTracker (Thermo Fisher Scientific) at 50 nM; cells were then fixed in 4% PFA in PBS. Images were acquired with a DMI6000 confocal microscope and Leica software with a 63x oil-immersion objective, with a zoom factor of 4, or with a Leica SP8X WLL microscope, equipped with a 405-nm laser, WLL2 laser (470–670 nm), and acousto-optical beam splitter. Images were acquired with a 63x1.4 objective. Samples were excited with a 405-nm laser and the emission recorded between 400 and 460 nm (ordered phase) and between 470 and 530 nm (disordered phase). Quantitative assessment of the membrane order was achieved by calculating the ratiometric relationship of the fluorescence intensity recorded in two spectral channels (GP value) by using an automated ImageJ macro, according to published guidelines (Owen et al., 2011). LysoTracker-positive regions were identified by thresholding the LysoTracker channel, and GP values were extracted from these regions using the macro cited above.

### Knockdown of target genes by RNAi in cells

SPHK2 or a scrambled control siRNA were purchased from Thermo Scientific Dharmacon (ONTarget plus siRNA and Control Pool). Cells were seeded in DMEM with 10% FBS. siRNA was diluted in siRNA buffer (final concentration 20 nM) before mixing with Lipofectamine 2000 for 20 min prior to being added to cells. To minimize Lipofectamine impact on the endolysosomal system, media was exchanged after 6 hr.

### SDS–PAGE and immunoblotting

Cells were pelleted by centrifugation followed by lysis in lysis buffer (10 mM Tris, 100 mM NaCl, 0.2% Triton X-100, 10 mM EDTA) on ice for 20 min. The lysates were transferred into fresh Eppendorf tubes and centrifuged (1000 × *g* for 1 min at 4°C) in a tabletop centrifuge to remove cellular debris. The protein concentration was determined using protein assay dye reagent concentrate (Bio-Rad). Proteins were separated under denaturing conditions by SDS–PAGE and transferred onto a PVDF membrane (Carl Roth) by standard wet blotting protocols. Samples were probed with rabbit polyclonal anti-SPHK2 (1:5000, 17096-1-AP ProteinTech) primary antibody. Mouse monoclonal anti-GAPDH antibody (1:5000, clone GAPDH-71.1, Sigma-Aldrich) was used as loading control. HRP-conjugated anti-mouse and anti-rabbit IgG secondary antibodies (Bio-Rad) were used for subsequent ECL-based detection (Bio-Rad).

### Fatty acid treatment in cells

For ALA, a stock solution was made in EtOH and stored in the dark at –20°C under a N_2_ atmosphere to prevent oxidation (Oliveira et al., 2015). PA was dissolved in 0.1 M NaOH according to Cousin et al., 2001. ALA and PA were then conjugated to BSA in 10% fatty acid-free solution by shaking for 1 hr at 37°C to a concentration of 6 mM for ALA and 5 mM for PA (Nůsková et al., 2023). Prior to pre-loading cells with BSA–PA, the solution was heated to 65°C for 15 min. For assays with C-Laurdan, cells were pre-loaded with FAs for 3 hr before the addition of C-Laurdan for another 2 hr. If pre-loading was followed by seeding with tau, pre-loading with FAs was done for 3 hr before fibrillar tau was added for an additional 5 hr. C-Laurdan was spiked in for the last 2 hr. No media changes were done between steps. For experiments with HEK293T tau-Venus cells and HEK293T sfGFP-LGALS3 cells, pre-loading with FAs was done for 3 hr before seeding with 1N4R tau fibrils. Cells were then fixed after 48 hr.

### Monomeric and fibrillar tau

Full-length human 1N4R tau was expressed and purified as previously described (Tardivel et al., 2016). The purified monomeric tau was dialyzed against PBS buffer containing 1 mM DTT, flash frozen in liquid nitrogen, and stored at –80°C. The concentration of monomeric tau was determined spectrophotometrically using an extinction coefficient at 280 nm of 7575 M^−1^ cm^−1^. Monomeric 1N4R tau was assembled into fibrils at 40 μM in the presence of 10 μM heparin, in PBS buffer containing 1 mM DTT under continuous shaking (600 rpm) for 5 days at 37°C in an Eppendorf Thermomixer. The quality of 1N4R tau fibrils produced after 5 days was assessed by Thioflavin T binding, sedimentation, and transmission electron microscopy (TEM). The resulting 1N4R tau fibrils were spun at 25°C and 75,000 × *g* for 30 min. The pelleted fibrils were resuspended in PBS buffer with 1 mM DTT at 50 μM equivalent monomeric tau concentration. 1N4R tau fibrils were labeled by incubation with 2 molar equivalents of lysine-reactive ATTO 550 (ATTO-TEC, GMBH) for 1 hr at room temperature. The unreacted fluorophore was removed by two cycles of centrifugation at 75,000 × *g* for 10 min and resuspension of the pellet in PBS. 1N4R tau fibrils were fragmented by sonication for 5 min in 2 ml Eppendorf tubes in a Vial Tweeter powered by an ultrasonic processor UIS250v (250 W, 2.4 kHz; Hielscher Ultrasonic, Teltow, Germany) to generate fibrillar particles with an average size of 50 nm as assessed by TEM analysis. For seeding experiments, either monomeric or fibrillar tau was added directly into the cell culture media to a final concentration of 400 nM. For C-Laurdan experiments, tau was added to cells for 5 hr with the last 2 hr C-Laurdan and LysoTracker were spiked into the media. For lysosomal rupture and tau seeding, cells were fixed after 48 hr with tau.

### Statistical analysis

Statistical analysis was performed with either GraphPad Prism (GraphPad Software, Version 6h and 10.1.1) or in R (version 4.3.1, with packages ‘nlme’ version 3.1.166, ‘lme4’ version 1.1-35.5, ‘emmeans’ version 1.10.6, ‘dplyr’ version 1.1.4). Data were assessed for normal distribution by Shapiro Normality Test where appropriate. Parametric or non-parametric tests were applied as indicated in the figure legends. Data presentation, sample size (number of replicates, and sample size per experiment and condition), and the applied statistical tests are indicated in the figure legends for each experiment. Sample size was chosen based on prior experience; no power calculation was used. Cells and animals to analyze were randomly selected from the sample dishes/plates. Unless otherwise indicated in the figure legends, cell culture data were collected in three independent experiments, with 10 images analyzed per experiment. For C-Laurdan and LysoTracker, the data were analyzed in R using a two-way mixed-model ANOVA to account for non-independence of measurements acquired from the same experiment and/or image. Models were fitted using the lme4 package. Pairwise comparisons between conditions were performed on estimated marginal means using the emmeans package with Sidak correction for multiple comparisons. Significance levels: non-significant (n.s.) p > 0.05, *p ≤ 0.05, **p ≤ 0.01, ***p ≤ 0.001, and ****p ≤ 0.0001.

## Data Availability

All data supporting the findings of this study are available within the paper and its supplementary information. *C. elegans* strains and plasmids generated in this study are available upon request.

## Additional files

Supplementary file 1. *C. elegans* strains used in this study.

Supplementary file 2. Main RNAi target genes of the bacterial clones used in this study.

MDAR checklist
