# Supplementary material for: Disruption of sphingolipid metabolism promotes tau seeding through endolysosomal membrane rigidification and rupture
Source: eLife. 2026 Aug 27;14:RP106865. doi: 10.7554/eLife.106865 (PMC13521564; doi:10.7554/eLife.106865)
Supplement: Supplementary file 1 [file elife-106865-fig3-figsupp1-data3.pdf]

SPHK2 Antibody

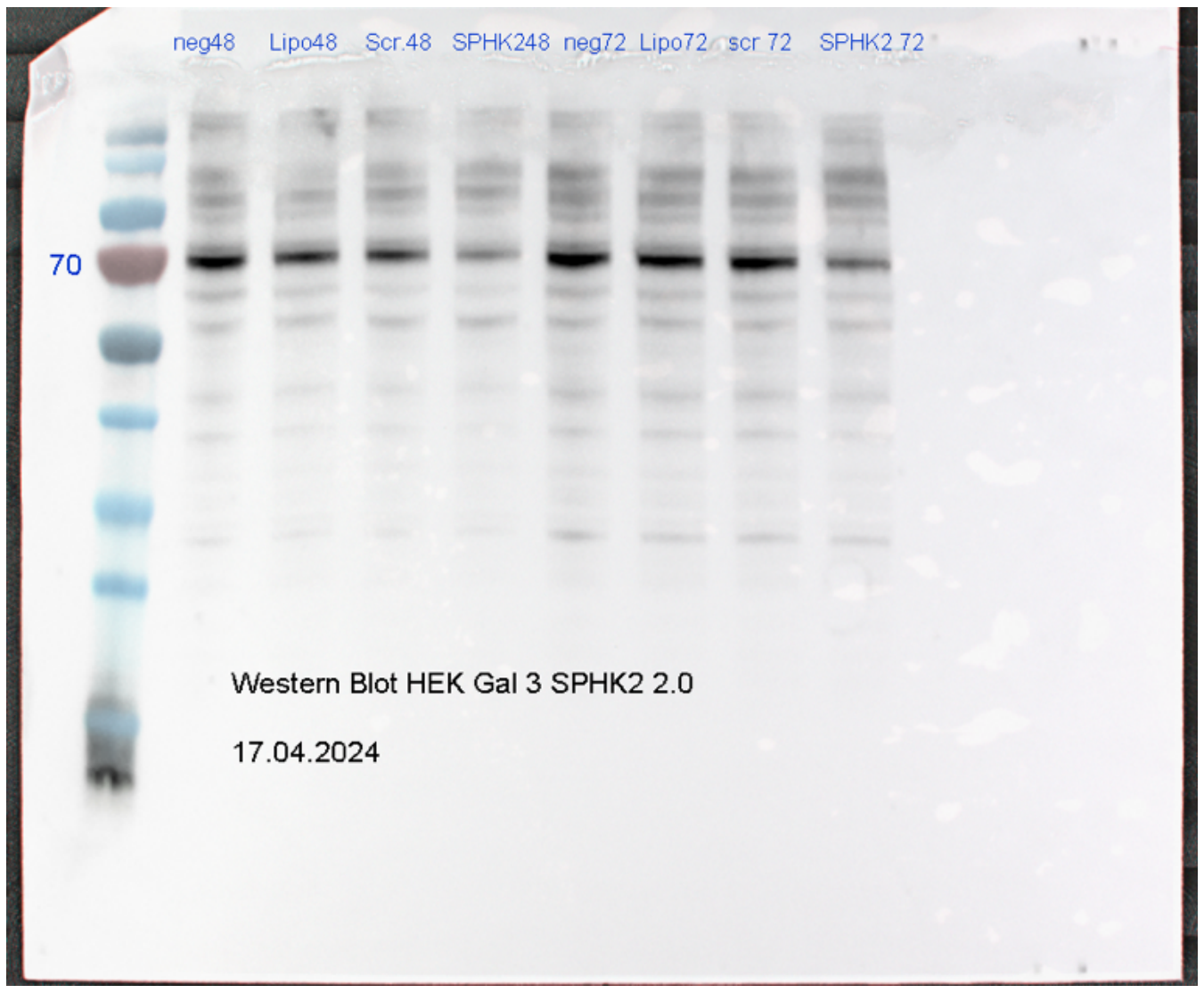

GAPDH Antibody

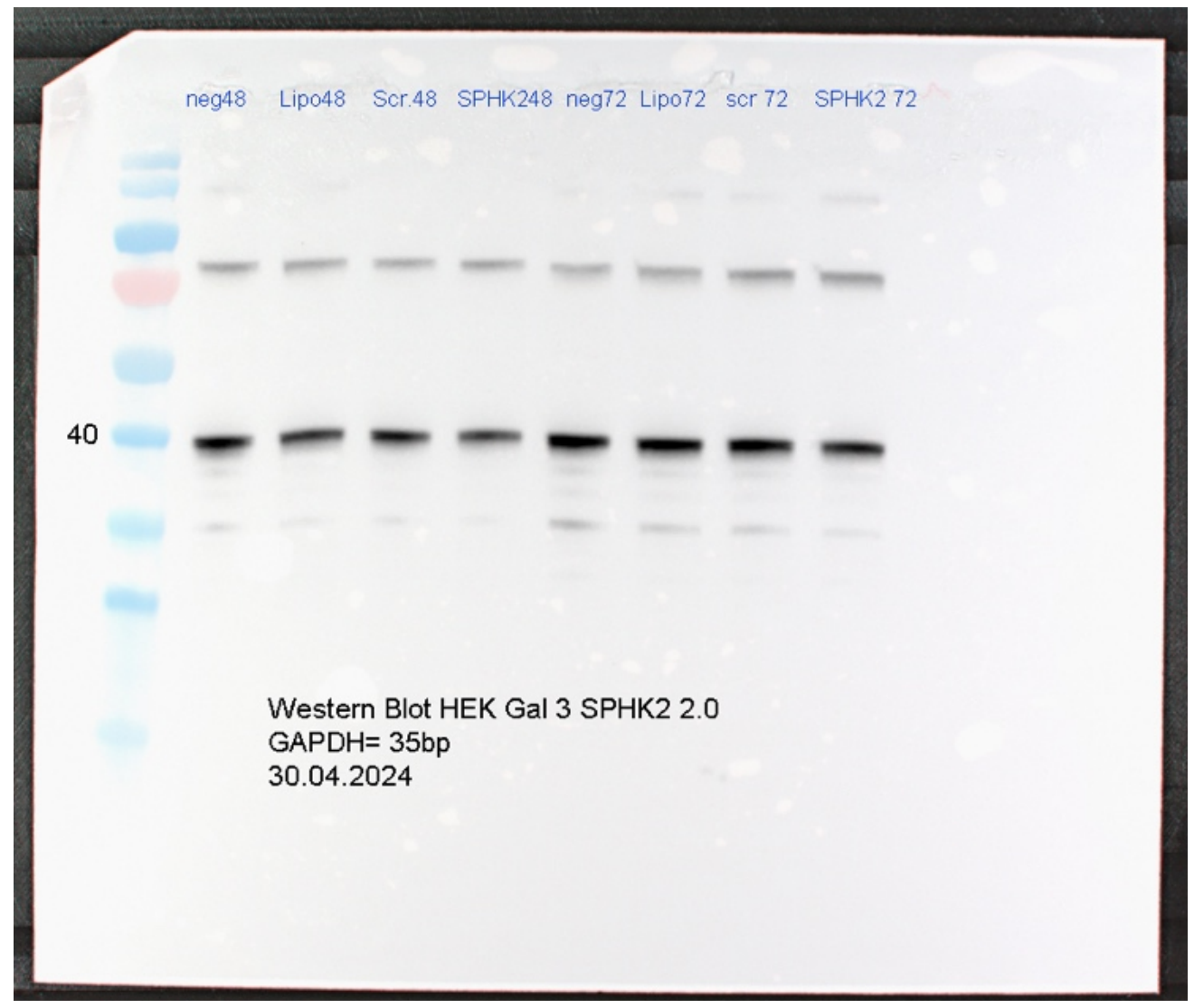

Figure Supplement 3, Source Data 1. Original membranes corresponding to Figure 3, panel C. Neg corresponds to untreated, Lipo corresponds to Lipofectamine, Scr corresponds to control (scrambled) siRNA.
