# Supplementary material for: Disruption of sphingolipid metabolism promotes tau seeding through endolysosomal membrane rigidification and rupture
Source: eLife. 2026 Aug 27;14:RP106865. doi: 10.7554/eLife.106865 (PMC13521564; doi:10.7554/eLife.106865)
Supplement: Peer review history

# Editor report: eLife Assessment

**DOI:** 10.7554/eLife.106865.3.sa0

**Authors:** Ellen AA Nollen

**Affiliations:** University of Groningen Netherlands

**Keywords:** Convincing, Important

This **important** study addresses the role of sphingolipid metabolism in maintaining endolysosomal membrane integrity and its impact on tau pathology in *Caenorhabditis elegans* and human cell culture models. The findings are **convincing**, and the proposed mechanisms are conceivable. The experimental evidence supports the conclusions of the study. The work will be of broad interest to cell biologists and biologists working on Alzheimer's disease and related proteinopathies.

# Referee report: Reviewer #1 (Public review)

**DOI:** 10.7554/eLife.106865.3.sa1

Summary:

In this study, Tittelmeier et al. explored the role of sphingolipid metabolism in maintaining endolysosomal membrane integrity and its downstream effects on tau aggregation and toxicity, using both worms and human cell models. The authors showed that knockdown of sphingolipid metabolism genes reduced endolysosomal membrane fluidity, as revealed by FRAP and C-Laurdan imaging, leading to increased vesicle rupture. Furthermore, tau aggregates accumulated in endolysosomes and exacerbated membrane rigidity and damage, promoting seeded tau aggregation, likely by enabling tau seed escape into the cytosol. Importantly, unsaturated fatty acid supplementation restored membrane fluidity, suppressed tau propagation, and alleviated neurotoxicity in *C. elegans*. These findings provide insight into how lipid dysregulation contributes to tau pathology and highlight membrane fluidity restoration as a potential therapeutic avenue for Alzheimer's disease.

Strengths:

The study addresses the connection between sphingolipid metabolism, endolysosomal membrane integrity, and tau pathology, which is a relevant topic in the context of Alzheimer's disease and related tauopathies.

The use of both *C. elegans* and human cell models provides cross-species perspectives that help frame the findings in a broader biological context.

The combination of FRAP and C-Laurdan dye imaging offers a biophysical approach to investigate changes in membrane properties, which is a technically interesting aspect of the study.

The observation that unsaturated fatty acid supplementation can modulate membrane fluidity and influence tau-related phenotypes adds an element of potential therapeutic interest.

The study presents multiple experimental approaches to address the proposed mechanism, and efforts were made to examine both membrane behavior and tau aggregation dynamics.

Comments on revised version:

I thank the authors for their thorough revisions and detailed responses. All of my previous concerns have been satisfactorily addressed, and I have no further comments.

# Referee report: Reviewer #2 (Public review)

**DOI:** 10.7554/eLife.106865.3.sa2

Tittelmeier et al. investigated the role of sphingolipid (SL) metabolism in the maintenance of endolysosomal vesicle integrity. They find that both impaired SL biosynthesis and degradation in *C. elegans* decreases the fluidity of endolysosomal membranes and promotes their rupture, while it has little effect on plasma membrane fluidity. Endolysosomal membrane fluidity is also negatively affected in human cells upon knockdown (KD) of a gene (SPHK2) involved in the SL degradation pathway. Aggregated forms of tau in both models (*C. elegans* and human cells) can also cause rigidification of the endolysosomal membrane, with SL homeostasis disruption having an additive effect, exacerbating endolysosomal rupture. Notably, KD of SPHK2 also increased the formation of tau foci, suggesting that compromised endolysosomal integrity may promote tau aggregation. These data provide a clearer understanding of how genetic manipulation of SL metabolism affects endolysosomal membranes and their rigidification in the context of tau aggregation. Supplementation of polyunsaturated fatty acids (PUFAs), which has a beneficial effect on Alzheimer's patients, improved membrane fluidity and reduced tau propagation in human cells and tau-associated neurotoxicity in *C. elegans*, suggesting a possible mechanism of action.

Comments on revised version:

The authors have:

Corrected editorial errors (Points 1 and 2).

Clarified the experimental rationale, added new data to rule out alternative explanation, and improved the presentation of the *C. elegans* model (Point 3).

Provided experimental evidence and appropriate discussion regarding the specificity and broader physiological context of SL gene knockdown effects (Point 4).

Overall, the authors' responses are thorough, supported by new data where appropriate, and demonstrate a clear understanding of the concerns raised. All points raised have been satisfactorily resolved.

# Referee report: Reviewer #3 (Public review)

**DOI:** 10.7554/eLife.106865.3.sa3

Summary:

The authors set off with an analysis of the lysosomal integrity upon knockdown of genes of the sphingolipid metabolic pathway that they identified in a previous work of an RNA screen using a new *C. elegans* Tau model. They then used cell culture and *C. elegans* experiments to study the link between lysosomal rupture and Tau propagation.

Strengths:

The authors use two complementary model systems and used probes to assess membrane rigidity that allow a quick assessment of the membrane dynamics and offer the opportunity to treat the cells with lipids, RNAi. Tau seeds etc.

Comments on revised version:

The authors have addressed the majority of my critical comments and thus I support the manuscript.

They have still not analysed the knockdown efficiencies of their RNAi experiments. But this is their choice.

The other publication establishing their Tau model is meanwhile published and there is no disconnect anymore between the model their analysis builds on.

# Author comment: Author response

**DOI:** 10.7554/eLife.106865.3.sa4

**Authors:** Jessica Tittelmeier, Carl Alexander Sandhof, Nicole Martin, Deike El-Kabarity, Soki-Bradel Ngonza-Nito, Ronald Melki, Carmen Nussbaum

**Affiliations:** Ludwig-Maximilians-Universität München Munich Germany; Massachusetts General Hospital Boston United States; Ludwig-Maximilians-Universität München Munich Germany; Ludwig-Maximilians-Universität München Munich Germany; CEA Paris-Saclay - Etablissement de Fontenay-aux-roses Fontenay-aux-Roses France; CEA-CNRS Fontenay-aux-Roses France; Ludwig-Maximilians-Universität München Munich Germany

The following is the authors’ response to the original reviews.

**Public Reviews:**

**Reviewer #1 (Public review):**

Summary:

In this study, Tittelmeier et al. explored the role of sphingolipid metabolism in maintaining endolysosomal membrane integrity and its downstream effects on tau aggregation and toxicity, using both worms and human cell models. The authors showed that knockdown of sphingolipid metabolism genes reduced endolysosomal membrane fluidity, as revealed by FRAP and C-Laurdan imaging, leading to increased vesicle rupture. Furthermore, tau aggregates accumulated in endolysosomes and exacerbated membrane rigidity and damage, promoting seeded tau aggregation, likely by enabling tau seed escape into the cytosol. Importantly, unsaturated fatty acid supplementation restored membrane fluidity, suppressed tau propagation, and alleviated neurotoxicity in *C. elegans*. These findings provide insight into how lipid dysregulation contributes to tau pathology and highlight membrane fluidity restoration as a potential therapeutic avenue for Alzheimer's disease.

Strengths:

The study addresses the connection between sphingolipid metabolism, endolysosomal membrane integrity, and tau pathology, which is a relevant topic in the context of Alzheimer's disease and related tauopathies.

The use of both *C. elegans* and human cell models provides cross-species perspectives that help frame the findings in a broader biological context.

The combination of FRAP and C-Laurdan dye imaging offers a biophysical approach to investigate changes in membrane properties, which is a technically interesting aspect of the study.

The observation that unsaturated fatty acid supplementation can modulate membrane fluidity and influence tau-related phenotypes adds an element of potential therapeutic interest.

The study presents multiple experimental approaches to address the proposed mechanism, and efforts were made to examine both membrane behavior and tau aggregation dynamics.

We thank the reviewer for this positive assessment of the study.

Weaknesses:

In Figure 3, the authors used C-Laurdan imaging to assess membrane fluidity and showed that knockdown of SPHK2, the human ortholog of sphk-1, led to increased membrane rigidity. However, the authors did not co-stain with a lysosomal marker, making it unclear whether the observed effect is specific to lysosomal membranes or reflects general membrane changes. Co-staining with LysoTracker or applying segmentation masks to isolate lysosomal signals would significantly improve interpretation.

We agree with the reviewer that it is important to isolate lysosomal signals for interpreting the C-Laurdan data. We therefore repeated and extended the C-Laurdan experiments in combination with LysoTracker staining and selectively analyzed LysoTracker-positive regions. These analyses showed pronounced increases in GP values in LysoTracker-positive vesicles after SPHK2 knockdown, supporting the conclusion that SPHK2 depletion increases endolysosomal membrane rigidity. We also performed LysoTracker-based analysis in the tau-fibril and fatty-acid experiments to better assess lysosome-associated membrane properties (see new and updated Figures 3B-E, Figures 5A and B, Figures S3A, B, F, G, Figures S4A-F, and Figures S5A-D for details). The respective Results sections have been revised accordingly.

Line 173 states that Lipofectamine 2000 increases membrane fluidity based on GP index changes, but this is incorrect. A higher GP index indicates increased membrane order (i.e., reduced fluidity), so the statement should be revised. Additionally, Lipofectamine 2000 can itself alter membrane rigidity, posing a risk of false-positive interpretations. To confirm the role of SPHK2 in this phenotype, the authors should use a CRISPR/Cas9 knockout model instead of relying solely on siRNA transfection, which may be confounded by the delivery reagent. Without lysosomal co-staining and SPHK2 KO validation, the authors cannot conclusively claim that SPHK2 loss affects endolysosomal membrane integrity.

We thank the reviewer for pointing out the incorrect wording regarding Lipofectamine. A higher GP index indicates increased membrane order/rigidity, not increased fluidity. Since the revised main figure now includes the SH-SY5Y data (Figure 3A-D), in which Lipofectamine alone did not significantly alter GP values (see Figure S3A, B), we removed the misleading statement from the Results.

We also agree that Lipofectamine can affect membrane properties and therefore needs to be carefully controlled. In all siRNA-mediated experiments, SPHK2 siRNA was compared to a matched control siRNA condition exposed to the same transfection reagent. We state in the Methods that cells were transfected with either SPHK2 or scrambled control siRNA and that the medium was exchanged after 6 h “to minimize lipofectamine impact on the endolysosomal system”. Thus, the effect attributed to SPHK2 KD is assessed relative to the appropriate Lipofectamine-containing control condition.

Importantly, we have now added lysosomal co-staining to address the reviewer’s concern about compartment specificity. This analysis showed that SPHK2 KD resulted in a pronounced increase in GP values within LysoTracker-positive compartments, demonstrating increased membrane rigidity at lysosomes. Thus, the revised data support the conclusion that SPHK2 KD increases lysosome-associated membrane rigidity, rather than only causing nonspecific effects on other cellular membranes.

We also clarified the relationship between the current siRNA-based assay and our previous CRISPR inhibition-based analysis. In the revised Results, we now write: “While SPHK2 KD alone significantly increased galectin puncta above the matched control, its effect was more modest than in our previous CRISPR inhibition-based analysis [20]. This difference likely stems from the earlier readout required for the combined siRNA/tau fibril assay, when transient Lipofectamine-associated effects still increased the control background.”

This addresses why the SPHK2 KD effect appears smaller in the current siRNA/tau-fibril assay than in our previous CRISPR inhibition-based analysis. The previous study, which is now peer-reviewed and published in the journal Autophagy, used a CRISPR inhibition-based strategy to reduce SPHK2 levels, which resulted in a highly significant increase in sfGFP-LGALS3 foci formation compared to the control [1]. Thus, the SPHK2 phenotype is not supported solely by the current siRNA experiment.

In addition, we sought to genetically validate the RNAi phenotypes using mutant strains. However, mutant strains were not available for all sphingolipid metabolism hits analyzed in this study. We therefore used the *sphk-1* mutant strain available at CGC (CZ24969; sphk-1(ju831)) to validate one of the key SL metabolism hits independently of RNAi. The revised manuscript states: “As genetic validation independent of RNAi, we tested an available sphk-1 mutant strain, which also showed a robust increase in hypodermal sfGFP::LGALS3 foci (Figure S1A).” This result supports the conclusion that genetic perturbation of sphingosine kinase activity compromises endolysosomal integrity in vivo.

Together, the revised manuscript addresses the reviewer’s concerns by correcting the GP interpretation, controlling the siRNA experiments against matched Lipofectamine-treated controls, adding LysoTracker-based lysosome-associated C-Laurdan analysis, relating the current siRNA data to our previous CRISPR inhibition-based analysis, and providing genetic validation for the available *sphk-1* mutant.

The section titled "Fibrillar tau increases membrane rigidity and exacerbates endolysosomal damage" (lines 177-215) requires substantial revision. The narrative jumps abruptly between worms and cell models, making it hard to follow the logic. The use of the F3ΔK281::mCherry strain is introduced without explanation or context. It is unclear whether this strain is relevant to lysosomal membrane rupture, as no reference or justification is provided. The authors should clarify whether this reporter is intended to detect lysosomal membrane permeabilization (LMP). If so, it would be more appropriate to use established LMP reporters, such as lysosome-targeted fluorescent sensors, galectin-based reporters, or dextran leakage assays. Based on the current data in Figure 3G, it is difficult to draw firm conclusions regarding membrane rupture levels.

We agree that this section required clarification, and we have substantially revised the Results to improve the logic and separation between model systems.

First, we now introduce the *C. elegans* reporter strain earlier in the manuscript, in the first Results section. In the revised text, we explain both the tau construct and the actual lysosomal damage reporter: “In this strain, endolysosomal membrane damage is monitored in the hypodermis by expression of human galectin-3 fused to superfolder-GFP (sfGFP::LGALS3). The animals also express an aggregation-prone tau fragment fused to mCherry (F3ΔK281::mCherry) in touch receptor neurons, which is transmitted to the hypodermis, as described previously [20].” We also clarify the principle of the Galectin reporter: “Under steady-state conditions, sfGFP::LGALS3 remains diffusely distributed throughout the cytosol. Upon endolysosomal damage, luminal β-galactosides become exposed and recruit sfGFP::LGALS3 into visible puncta, providing a sensitive readout of vesicle rupture.” Thus, F3ΔK281::mCherry is not the reporter for lysosomal membrane permeabilization; the membrane-damage readout is sfGFP::LGALS3 puncta formation.

Second, we reorganized the manuscript to separate the human cell experiments from the *C. elegans* experiments more clearly. The revised section “Fibrillar tau and SPHK2 KD act in concert to exacerbate endolysosomal damage and seeded tau aggregation” now focuses on human cell data. The *C. elegans* experiments are now presented in a separate section, “Tau transmission sensitizes endolysosomal membranes to sphingolipid perturbations in vivo.” We believe that this revised structure now clearly distinguishes the role of the Galectin reporter from the tau transmission model, separates the human cell and *C. elegans* data, and avoids the abrupt transitions between model systems noted by the reviewer.

To support the conclusion that sphingolipid metabolism gene knockdown alters membrane properties, the study would benefit from direct lipidomic analysis. Measuring changes in sphingolipid profiles in both *C. elegans* and cell models would provide biochemical evidence for the proposed disruption of lipid homeostasis. Given the availability of lipidomics platforms, this type of analysis should be feasible in both worms and human cells and would significantly strengthen the mechanistic claims regarding membrane fluidity and integrity.

Because we did not perform lipidomics in the present study, we have revised the wording throughout the manuscript to avoid implying that we directly measured lipid composition. Instead, we now refer to “genetic perturbation/disruption of sphingolipid metabolism” or “knockdown of enzymes involved in sphingolipid metabolism” when describing our experimental interventions.

We agree that lipidomic analyses will be important in future studies to define how perturbation of sphingolipid metabolism changes lipid composition in *C. elegans* and human cells. However, lipidomics itself would not directly establish which lipid changes causally drive the membrane rigidification observed in our study. Membrane fluidity is a biophysical property determined by the combined composition of the membrane, including lipid abundance, saturation, acyl-chain length, head groups, sterol content, and membrane-associated proteins. Thus, even if lipidomics identified changes in sphingolipid profiles, these changes could not be directly translated into a predictable effect on membrane fluidity without additional biophysical validation, using Laurdan dye imaging or FRAP. Moreover, whole-cell or whole-animal lipidomics would not resolve whether the relevant lipid changes occur specifically at endolysosomal membranes, which are the focus of our study.

We have now clarified this point in the Discussion. Specifically, we state that “even detailed lipidomics would not by itself identify which lipid changes are responsible for the observed membrane rigidification” and that future lysosome-enriched or organelle-specific lipidomic approaches should be combined with direct manipulation of candidate lipid species, followed by measurements of membrane fluidity and rupture, to determine which lipid changes causally contribute to endolysosomal membrane rigidification. In the present study, we therefore focused on direct quantitative biophysical readouts of membrane properties in *C. elegans*. We used FRAP of the lysosomal membrane protein LAAT-1::mCherry to assess lateral mobility within lysosomal membranes and showed that knockdown of sphingolipid-metabolism genes increased the time to half-maximal recovery, indicating reduced lysosomal membrane fluidity. Notably, knockdown of genes involved in both sphingolipid biosynthesis and sphingolipid degradation increased membrane rigidity. This makes it unlikely that the observed rigidification is caused by accumulation or depletion of a single shared lipid species. Rather, perturbations at different steps of sphingolipid metabolism may lead to distinct lipidomic changes that nevertheless converge on a common biophysical outcome: reduced endolysosomal membrane fluidity. In parallel, we used C-Laurdan imaging to quantify membrane order and found that SPHK2 knockdown in SH-SY5Y human neuroblastoma cells increased GP values, consistent with increased membrane rigidity. Two-channel thresholding of LysoTracker-positive compartments further showed that SPHK2 knockdown increased GP values in lysosome-associated regions.

Thus, although lipidomics will be valuable to define the underlying lipid changes in future work, the current data already provide convergent quantitative evidence from independent membrane-fluidity readouts across *C. elegans* and human cell models. This cross-model consistency strengthens the robustness and reproducibility of the central conclusion that perturbation of sphingolipid metabolism alters endolysosomal membrane properties and promotes membrane rupture.

The conclusions of the study rely heavily on imaging-based assays, including FRAP, C-Laurdan, and fluorescence microscopy. While these approaches provide valuable spatial and qualitative insights, they are inherently indirect and subject to interpretive limitations. To strengthen the mechanistic claims, the authors should incorporate additional biochemical or quantitative approaches. For example, lipidomics would allow direct measurement of membrane lipid composition changes, and western blotting or quantitative proteomics could assess levels of membrane-associated proteins involved in endolysosomal function or stress responses. Including such data would significantly improve the robustness and reproducibility of the study's conclusions.

We agree that lipidomic and proteomic analyses will be important in future studies to define which sphingolipid species and/or membrane-associated proteins contribute to the observed rigidification of endolysosomal membranes. In response to this point, we have revised the wording throughout the manuscript to more precisely distinguish our experimental interventions from inferred changes in lipid composition. Because we did not directly measure lipid composition in the present study, we now refer more specifically to “genetic perturbation/disruption of sphingolipid metabolism” or “knockdown of enzymes involved in sphingolipid metabolism” when describing our data, rather than implying that global sphingolipid homeostasis was directly quantified. We retain “sphingolipid imbalance” only in interpretive or model-based statements where appropriate.

However, we respectfully disagree that the current data are only qualitative. FRAP and C-Laurdan GP imaging are established quantitative biophysical approaches: FRAP provides quantitative parameters such as the time to half-maximal recovery and the mobile fraction, whereas C-Laurdan GP provides a ratiometric measurement of membrane lipid order and packing. Similarly, the Galectin puncta assay is an established quantitative readout of lysosomal membrane permeabilization. Thus, while these approaches are imaging-based, they provide quantitative readouts of membrane mobility, membrane order, and membrane rupture, respectively.

We also note that lipidomic and proteomic profiling, although valuable, would not by itself establish which lipid or protein changes causally drive the membrane rigidification observed in our study. Membrane fluidity is an emergent biophysical property determined by the combined composition of the membrane, including lipid abundance, saturation, acyl-chain length, head groups, sterol content, and membrane-associated proteins. Therefore, an increase or decrease in a given lipid or protein species cannot be directly translated into a predictable change in membrane fluidity without additional biophysical validation. This point is further supported by our observation that knockdown of genes involved in both sphingolipid biosynthesis and sphingolipid degradation increased endolysosomal membrane rigidity. These perturbations would be expected to affect lipid composition in different, possibly even opposing, ways, making it unlikely that the shared rigidification phenotype is caused by accumulation or depletion of one single lipid species. Rather, distinct lipidomic changes may converge on a common biophysical outcome: reduced endolysosomal membrane fluidity.

We have clarified this point in the Discussion and now state that future lysosome-enriched or organelle-specific lipidomic/proteomic approaches should be combined with direct manipulation of candidate lipid or protein species, followed by measurements of membrane fluidity and rupture, to determine which changes causally contribute to endolysosomal membrane rigidification. Such experiments would address the distinct question of which molecular components mediate the effect. By contrast, the central aim of the present study was to test whether genetic perturbation of enzymes involved in sphingolipid metabolism alters membrane fluidity and thereby promotes endolysosomal rupture and tau seeding.

For this question, direct biophysical measurements of membrane fluidity and quantitative readouts of membrane rupture are the most relevant assays. We therefore used complementary quantitative approaches in two distinct model systems: FRAP of the lysosomal membrane protein LAAT-1::mCherry in *C. elegans* and C-Laurdan GP imaging in human cells. The fact that perturbing sphingolipid metabolism reduced endolysosomal membrane fluidity in *C. elegans* and increased lysosome-associated membrane rigidity in human cells supports the robustness and reproducibility of the central conclusion across independent model systems. In the revised manuscript, we further strengthened the human-cell data by adding SH-SY5Y neuroblastoma cells as a neuronal-like model and by combining C-Laurdan imaging with LysoTracker-based analysis to assess lysosome-associated membrane properties.

To further address causality, we manipulated membrane fluidity independently of sphingolipid metabolism enzymes using fatty acid supplementation. Increasing membrane rigidity with PA exacerbated tau-induced endolysosomal rupture and seeded aggregation, whereas increasing membrane fluidity with ALA reduced tau-induced membrane rigidification, endolysosomal rupture, and seeded aggregation. Thus, the revised manuscript combines genetic perturbation of sphingolipid metabolism, quantitative membrane-fluidity measurements, whole-cell and lysosome-associated C-Laurdan analysis, and Galectin-based rupture assays across complementary models.

Regarding lysosomal function, we agree that functional readouts are informative, but lysosomal membrane rupture and global lysosomal degradative capacity are related but not identical readouts. This distinction is supported by Yong et al., who reported that lipid dysregulation can induce lysosomal membrane permeabilization and lysosomal accumulation of endogenous protein aggregates without broadly impairing core lysosomal or proteasomal functions [2]. Accordingly, the absence of overt defects in general lysosomal activity would not necessarily exclude membrane damage.

The human cell experiments were performed exclusively in HEK293T cells, which are not physiologically relevant for modeling Alzheimer's disease or lysosomal function in neurons. Given that the study aims to draw conclusions related to tau aggregation and lysosomal membrane integrity, the use of a more disease relevant cellular model is essential. There are several established AD-relevant cell models, including iPSCderived neurons, neuroblastoma lines expressing tau, or microglial models, which would better reflect the cellular context of tauopathies. Validation of key findings in at least one of these systems would substantially enhance the biological relevance and translational impact of the study.

We have expanded and clarified the human cell data in the revised manuscript. Specifically, we now include SH-SY5Y human neuroblastoma cells for key C-Laurdan experiments assessing membrane rigidity after SPHK2 knockdown. We also show that recombinant tau fibrils increased membrane rigidity in SHSY5Y and HEK293T cells, including in LysoTracker-positive compartments.

Importantly, the HEK293T cells are used for specific, established quantitative assays rather than as a model of neuronal toxicity. In particular, HEK293T sfGFP-LGALS3 cells are used to quantify galectin puncta formation as a readout of endolysosomal rupture, and HEK tau-Venus biosensor cells are used to quantify seeded tau aggregation. Thus, SH-SY5Y cells and HEK293T cells are used for complementary purposes: SHSY5Y cells provide a more neuronal-like human cell context for membrane-rigidity measurements, whereas HEK293T reporter/biosensor cells provide robust quantitative assays for galectin puncta formation and tau seeding.

In addition, tau-associated neuronal dysfunction and toxicity were assessed in vivo, in functional *C. elegans* touch receptor neurons. In the revised manuscript, we show that ALA supplementation mitigated the age-dependent touch-response deficit and reduced neurotoxicity in animals expressing F3ΔK281::mCherry in touch receptor neurons. We have also revised the wording throughout the manuscript to avoid implying that HEK293T cells are used to model neuronal toxicity.

Finally, the relevance of these hits to human neuronal tau seeding is also supported by our previous study, in which conserved hits from the *C. elegans* screen, including sphingosine kinase perturbation, were validated in human iPSC-derived neurons for their effect on seeded tau aggregation [1]. We now cite this published study where appropriate. Together, the revised manuscript combines neuronal-like human SH-SY5Y cells, established HEK293T tau-seeding and galectin reporter assays, in vivo neuronal readouts in *C. elegans*, and prior validation in human iPSC-derived neurons, thereby strengthening the biological relevance of the conclusions while using each model for the assay in which it is most informative.

The authors reported that PUFA supplementation rescues neurotoxic phenotypes by increasing membrane fluidity. However, the data supporting this claim rely entirely on confocal imaging, shown in both the main and supplemental figures. To substantiate the mechanistic link between PUFA treatment and improved lysosomal membrane properties, the authors should include functional assays demonstrating that PUFAs are indeed incorporated into lysosomal membranes. Additionally, lipidomics analysis would be valuable to identify which lipid species are altered upon supplementation and correlate these changes with the observed phenotypic rescue. Furthermore, the conclusion that PUFAs rescue "neurotoxic phenotypes" is not appropriate based on data derived solely from HEK293T cells, which are not neuronal. To make claims about tau-related neurotoxicity, the authors should validate their findings in a more relevant neuronal model, such as SH-SY5Y neuroblastoma cells expressing tau or iPSC-derived neurons. This would better reflect the cellular environment of Alzheimer's disease and provide stronger support for the proposed therapeutic potential of PUFA supplementation.

We agree that PUFA supplementation can have effects beyond membrane fluidity and that our data do not directly demonstrate incorporation of ALA into lysosomal membranes. We have therefore revised the Discussion to explicitly acknowledge this limitation. In the revised text, we state that “PUFAs can also influence lipid signaling, oxidative stress responses, and broader membrane remodeling” and that we “cannot exclude additional direct or indirect effects of ALA.” At the same time, we note that the opposing effects of PA and ALA, together with the sphingolipid-metabolism knockdown data, support membrane fluidity as a major determinant of endolysosomal membrane integrity and rupture in our models. To strengthen the link between ALA and lysosome-associated membrane properties, we combined CLaurdan imaging with LysoTracker-based analysis. In the revised Results, we show that ALA prevented tau-induced membrane rigidification and that LysoTracker-based analysis indicated effects on lysosome-associated membrane properties. ALA also reduced tau-induced endolysosomal rupture and seeded aggregation in human cell models.

Regarding lipidomics, we refer to our response above and to the revised Discussion. We agree that lipidomics would be valuable to identify ALA-induced lipid changes, but such data would not by itself establish how these changes affect membrane fluidity without additional biophysical validation.

Finally, we clarify that our conclusion regarding tau-associated neuronal dysfunction and toxicity is not based on HEK293T cells. HEK293T cells were used for established quantitative assays of Galectin puncta formation and seeded tau aggregation. The neurotoxicity experiments were performed in vivo in *C. elegans* touch receptor neurons, where ALA supplementation reduced galectin foci formation, mitigated age-dependent touch-response deficit and reduced neuronal toxicity.

While the authors demonstrate that ALA supplementation mitigates neurotoxicity in *C. elegans* expressing aggregated tau (F3ΔK281::mCherry), the current data are not sufficient to conclude that ALA directly rescues tau aggregation toxicity via a lysosome-specific mechanism. It remains unclear how lipid composition is altered upon ALA treatment and whether these changes correlate with functional improvement of lysosomal pathways. The manuscript does not provide mechanistic insight into how ALA enhances lysosomal health or attenuates endolysosomal damage. Moreover, supplementation with PUFAs like ALA can activate a wide range of cellular processes beyond lysosomal function, including alterations in membrane fluidity, signaling cascades, and oxidative stress responses. The authors should clarify how they distinguish the lysosome-related effects from these alternative pathways. For example, did they observe specific lysosomal markers or structural improvements in lysosomes upon ALA treatment?

Additional data or controls would be necessary to support a lysosome-specific protective mechanism and to exclude the involvement of other PUFA-responsive pathways in the observed phenotypes.

We agree that our data do not prove that ALA acts exclusively through a lysosome-specific mechanism or that ALA is directly incorporated into lysosomal membranes. We have therefore revised the manuscript to avoid this interpretation and explicitly acknowledge alternative PUFA-responsive pathways. In the revised Discussion, we state that “PUFAs can also influence lipid signaling, oxidative stress responses, and broader membrane remodeling” and that we “cannot exclude additional direct or indirect effects of ALA.” We further conclude more cautiously that the opposing effects of PA and ALA, together with the sphingolipid metabolism perturbation data, support membrane fluidity as a major determinant of endolysosomal membrane integrity and rupture in our models.

To strengthen the lysosome-related aspect of the mechanism, we added LysoTracker-based analysis to the C-Laurdan experiments. In the revised Results, ALA prevented tau-induced membrane rigidification, and LysoTracker-based analysis indicated that ALA also affected lysosome-associated membrane properties. ALA further reduced tau-induced Galectin puncta formation and seeded tau aggregation in human cell models. These data support an effect of ALA on lysosome-associated membrane order and rupture, while not excluding additional effects through lipid signaling, oxidative stress responses, or other PUFA-responsive pathways.

Regarding lipid composition, we refer to the revised Discussion and our response above. We agree that lipidomics would be valuable to identify ALA-induced lipid changes, but such analyses would need to be organelle-specific and combined with biophysical validation to determine how candidate lipid changes affect membrane fluidity and rupture.

Finally, we clarify that our conclusion regarding tau-associated neuronal dysfunction and toxicity is based on the *C. elegans* experiments, not on HEK293T cells. HEK293T cells were used for quantitative Galectin puncta and tau-seeding assays, whereas neuronal dysfunction and toxicity were assessed in vivo in touch receptor neurons in *C. elegans*. In the revised Results, we state that ALA supplementation mitigated galectin foci formation, age-dependent touch-response deficit and reduced neuronal toxicity in animals expressing F3ΔK281::mCherry in these neurons.

**Reviewer #2 (Public review):**

Tittelmeier et al. investigated the role of sphingolipid (SL) metabolism in the maintenance of endolysosomal vesicle integrity. They find that both impaired SL biosynthesis and degradation in *C. elegans*, decrease the fluidity of endolysosomal membranes and promote their rupture, while it has little effect on plasma membrane fluidity. Endolysosomal membrane fluidity is also negatively affected in human cells upon knockdown (KD) of a gene (SPHK2) involved in the SL degradation pathway. Aggregated forms of tau in both models (*C. elegans* and human cells) can also cause rigidification of the endolysosomal membrane, with SL homeostasis disruption having an additive effect, exacerbating endolysosomal rupture. Notably, KD of SPHK2 also increased the formation of tau foci, suggesting that compromised endolysosomal integrity may promote tau aggregation. These data provide a clearer understanding of how genetic manipulation of SL metabolism affects endolysosomal membranes and their rigidification in the context of tau aggregation. Supplementation of polyunsaturated fatty acids (PUFAs), which has a beneficial effect on Alzheimer's patients, improved membrane fluidity and reduced tau propagation in human cells and tau-associated neurotoxicity in *C. elegans*, suggesting a possible mechanism of action.

Overall, the conclusions of this paper are supported by the data, with a few aspects requiring further clarification and elaboration.

(1) A reference to Figure S2E-G, which shows that KD of SL biosynthesis genes do not affect the plasma membrane, is missing from the main text.

We thank the reviewer for pointing this out. We have added the reference to the respective figures in the main text when discussing the plasma membrane FRAP experiments.

(2) In Figure 3C, lipofectamine alone shows that it increases membrane rigidity (increased GP values), not membrane fluidity.

We thank the reviewer for pointing out this incorrect wording. A higher GP index indicates increased membrane order/rigidity, not increased membrane fluidity. Since the revised main figure now includes the SH-SY5Y data, in which Lipofectamine alone did not significantly alter GP values, we removed the misleading statement from the Results. Importantly, all siRNA-mediated knockdown experiments were compared to matched control siRNA conditions exposed to the same transfection reagent. Thus, the effect attributed to SPHK2 KD is assessed relative to the appropriate Lipofectamine-containing control condition.

(3) In Figure 3F, the EV cntl condition expressing F3:mCh tau should have increased LGALS3 foci compared to the mCh EV cntl according to Ref (20) and its Figure 2G (at least for Day 5 animals), which would be indicative of the tau spreading in hypodermal tissue. What *C. elegans* age was examined in Figure 3F? Can the authors provide evidence of the transmission of the F3:mCh tau from the touch receptor neurons to the hypodermis in the EV [similar to Figure 2C & D from Ref (20)] and compare it to the KDs? Otherwise, it seems that KD of SL genes impacts not only endolysosomal rupture but significantly affects tau accumulation/spreading as well (e.g., shown later in HEK cells, where SPHK2 KD increases the formation of tau-Venus foci).

We thank the reviewer for raising this important point. The analysis referred to by the reviewer has now been moved to the revised *C. elegans* section and is presented as Figure 4A and B. The experiments were performed in the reporter strain used in our genome-wide screen In Ref (20), now published in Autophagy [1]. We clarified the purpose of the reporter strain and the relationship between tau transmission and the galectin puncta readout. In the revised manuscript, we now state: “In this strain, endolysosomal membrane damage is monitored in the hypodermis by expression of human galectin-3 fused to superfolder-GFP (sfGFP::LGALS3). The animals also express an aggregation-prone tau fragment fused to mCherry (F3ΔK281::mCherry) in touch receptor neurons, which is transmitted to the hypodermis, as described previously [20].” We further clarify that sfGFP::LGALS3 puncta formation, not F3ΔK281::mCherry, is the readout of endolysosomal rupture: “Under steady-state conditions, sfGFP::LGALS3 remains diffusely distributed throughout the cytosol. Upon endolysosomal damage, luminal β-galactosides become exposed and recruit sfGFP::LGALS3 into visible puncta, providing a sensitive readout of vesicle rupture”.

Furthermore, we now better explain that transmitted tau sensitizes endolysosomal membranes to additional perturbations rather than necessarily inducing a strong lysosomal rupture phenotype on its own. In the experiments shown in Figure 4A and B, we compare F3ΔK281::mCherry animals with matched mCherry-only control animals that also express sfGFP::LGALS3 in the hypodermis. We now state: “We compared animals expressing F3ΔK281::mCherry in touch receptor neurons, from where it is transmitted to the hypodermis, with matched controls expressing mCherry alone in the same neurons. In both strains, sfGFP::LGALS3 is expressed in the hypodermis to monitor endolysosomal membrane damage.” We have also clarified the age of the animals in the revised figure legends.

To experimentally address whether the enhanced rupture phenotype could be explained by altered tau transmission, we quantified hypodermal F3ΔK281::mCherry levels after *sphk-1* RNAi (new Figure 4C, D). Importantly, *sphk-1* RNAi did not increase hypodermal F3ΔK281::mCherry levels, arguing that the enhanced rupture phenotype is not due to increased tau transmission. Moreover, *C. elegans* neurons are largely refractory to systemic RNAi under the conditions used here [3]. We have added this important information to the Discussion. Specifically, the revised manuscript states that “the enhanced rupture phenotype is unlikely to result from a direct effect of RNAi on neuronal F3ΔK281::mCherry expression, as *C. elegans* neurons are largely refractory to systemic RNAi under the conditions used here,” supporting the interpretation that the RNAi treatments primarily affect endolysosomal integrity in the recipient tissue rather than neuronal tau expression itself.

Finally, we would like to clarify that the increased tau-Venus foci in HEK cells should not be interpreted as a direct induction of tau aggregation by SPHK2 KD. Only upon addition of recombinant tau fibrils did SPHK2 KD significantly increase tau-Venus foci formation (Figure 3 H, I). This is consistent with the control experiments performed in human iPSCs in our previous study and supports our interpretation that perturbation of sphingolipid metabolism increases susceptibility to seeded tau aggregation by promoting endolysosomal rupture and tau seed escape, rather than by directly increasing tau aggregation or tau transmission.

(4) Sphingolipids are essential membrane components and signaling molecules. Does KD of SL genes in *C. elegans* and the subsequent endolysosomal rupture cause any major, intermediate, or minor defects/phenotypes (in non-aggregation prone models, w/t.)?

We agree that sphingolipids are essential membrane components and signaling molecules and that perturbing sphingolipid metabolism can have broader physiological consequences. In the revised manuscript, we address this point in two ways.

First, we directly tested whether SL gene knockdown can induce endolysosomal rupture independently of aggregation-prone tau by using matched control animals expressing mCherry alone in touch receptor neurons while also expressing sfGFP::LGALS3 in the hypodermis. In these animals, knockdown of most SL-related hits resulted in nearly all animals displaying hypodermal sfGFP::LGALS3 foci, indicating that perturbation of SL metabolism can compromise endolysosomal integrity in the absence of transmitted F3ΔK281::mCherry (Figure 4A, B).

Second, we have added a Discussion paragraph to place these findings into a broader physiological context. We now clarify that endolysosomal membrane rupture and global lysosomal function are related but not identical readouts. In support of this distinction, we discuss work showing that lipid dysregulation can induce lysosomal membrane permeabilization and lysosomal accumulation of endogenous protein aggregates without broadly impairing core lysosomal or proteasomal function [2]. Thus, membrane damage can occur even when general lysosomal activity is not overtly disrupted.

We also discuss a recent study published during the revision of this manuscript that independently identified SPHK-1 as an important regulator of lysosomal integrity in *C. elegans*, showing that strong *sphk1* loss-of-function causes lysosomal sphingosine accumulation, membrane rupture, impaired degradative function, cargo accumulation, developmental defects, and reduced lifespan [4].

Importantly, while that study focused on a strong loss-of-function mutation in a single SL-metabolism gene, our data show that knockdown of multiple SL-metabolism genes, including genes involved in both SL biosynthesis and degradation, converges on reduced endolysosomal membrane fluidity and increased rupture. This suggests that the observed membrane rigidification and rupture are not specific to one mutant background but represent a broader consequence of perturbing SL metabolism at multiple points. A systematic characterization of all organismal phenotypes caused by each SL gene knockdown was beyond the scope of the present study. Therefore, the revised manuscript now makes clear that the study focuses on endolysosomal membrane fluidity and rupture because these membrane-level changes are directly linked to tau seed escape and seeded tau aggregation, while broader physiological consequences may vary depending on the strength and context of the perturbation.

**Reviewer #3 (Public review):**

Summary:

The authors set off with an analysis of the lysosomal integrity upon knockdown of genes of the sphingolipid metabolic pathway that they identified in a previous (yet unpublished) work of an RNA screen using a new *C. elegans* Tau model. They then used cell culture and *C. elegans* experiments to study the link between lysosomal rupture and Tau propagation.

Strengths:

The authors use two complementary model systems and use probes to assess membrane rigidity that allow a quick assessment of the membrane dynamics and offer the opportunity to treat the cells with lipids, RNAi. Tau seeds, etc.

Weaknesses:

The main weakness is that this work builds on not-yet-peer-reviewed manuscript that established a new *C. elegans* Tau model and RNAi screen that aimed to identify genes involved in the propagation of Tau.

This reviewer misses essential information of the *C. elegans* Tau strain (not included in the method section): e.g., promoter used for the expression, information on the used Tau variant, expression pattern, and aggregation, etc.

We thank the reviewer for raising this point. The related study establishing the *C. elegans* tau transmission model and RNAi screen has now been peer-reviewed and published in Autophagy [1]. We now cite the published article throughout the revised manuscript instead of the previous preprint.

We also agree that the current manuscript should be understandable without requiring the reader to consult the previous paper for the basic logic of the model. We therefore added a clearer introduction of the reporter strain in the Results. Specifically, we now explain that the strain expresses the aggregation-prone tau fragment F3ΔK281::mCherry in touch receptor neurons, that this tau fragment is transmitted to the hypodermis, and that endolysosomal membrane damage is monitored in the hypodermis using sfGFP::LGALS3. We further clarify that sfGFP::LGALS3 remains diffuse under steady-state conditions and forms puncta upon endolysosomal membrane damage, when luminal β-galactosides become exposed. Thus, the revised manuscript now provides the key information needed to understand the experimental system used here, while the published Autophagy paper is cited for the full characterization of the tau transmission model, expression pattern, aggregation properties, and original genome-wide RNAi screen.

Throughout the study, I missed data on:

(1) Effect of the knockdown on Tau expression, localisation (with lysosomal membrane?), aggregation, and proteotoxicity. The effect of the RNAi-mediated knockdown could also simply lead to a reduced expression of Tau that, in turn, leads to suppressed propagation.

We agree that it is important to distinguish effects on tau expression/transmission from effects on endolysosomal membrane integrity. In the *C. elegans* experiments, F3ΔK281::mCherry is expressed in touch receptor neurons and transmitted to the hypodermis, where sfGFP::LGALS3 reports endolysosomal membrane damage. We now describe this more clearly in the revised Results.

The RNAi treatments target genes involved in sphingolipid metabolism under systemic RNAi conditions. Because *C. elegans* neurons are largely refractory to systemic RNAi in the absence of sensitizing backgrounds [3], which we did not use, a direct RNAi-mediated reduction of neuronal F3ΔK281::mCherry expression is very unlikely. We have added this point to the Discussion, stating that “the enhanced rupture phenotype is unlikely to result from a direct effect of RNAi on neuronal F3ΔK281::mCherry expression, as *C. elegans* neurons are largely refractory to systemic RNAi under the conditions used here.”

Experimentally, we also tested whether sphingolipid perturbation alters transmitted tau levels (new Figure 4C, D). Specifically, we quantified hypodermal F3ΔK281::mCherry after *sphk-1* RNAi and found no increase, arguing that the enhanced rupture phenotype is not due to increased tau transmission.

Moreover, in the cell-based tau-Venus assay, SPHK2 knockdown alone did not induce detectable tau aggregation in the absence of exogenously added tau fibrils (Figure 3H, I). Only upon addition of recombinant tau fibrils did SPHK2 knockdown significantly increase tau-Venus foci formation. We now state this explicitly in the revised Results and conclude that disruption of sphingolipid metabolism is not sufficient on its own to initiate detectable tau aggregation under the conditions tested here but rather increases cellular susceptibility to seeded tau aggregation when tau fibrils are present.

Together, these data argue against a direct effect of sphingolipid gene knockdown on tau expression or spontaneous tau aggregation. Instead, they support our interpretation that perturbation of sphingolipid metabolism compromises endolysosomal membrane integrity, thereby facilitating tau seed escape and seeded aggregation when tau seeds are present.

(2) A quantification of RNAi knockdown is needed to judge the efficiency of the RNAi, in particular for the combinatorial RNAi experiments involving 2 and even 4 genes in parallel. Ideally, these analyses should be validated with mutants for these genes.

We agree that RNAi efficiency can vary between clones and that this is particularly relevant for combinatorial RNAi experiments targeting two or more potentially redundant genes. We have added this limitation to the Results section and now state: “Because KD efficiency was not assessed for the individual RNAi clones or co-RNAi combinations, these experiments do not allow comparison of relative RNAi strength or inference of the relative importance of individual genes. Thus, the conclusions drawn from these RNAi experiments are qualitative: specific single or combined KDs can promote endolysosomal rupture, whereas the absence of a detectable phenotype after RNAi cannot exclude gene involvement, as KD may have been insufficient.”

Where mutant strains were available, we performed genetic validation. Specifically, a *sphk-1* mutant available at CGC (CZ24969; *sphk-1*(*ju831*)) also showed increased hypodermal sfGFP::LGALS3 puncta (new Figure S1A), supporting the RNAi-based conclusion that genetic perturbation of sphingolipid metabolism compromises endolysosomal integrity. Corresponding mutant strains were not available for the other selected hits. Importantly, most hits also induced sfGFP::LGALS3 foci in human HEK293T cells as assessed in our previous study [1], providing additional support that the observed effects are not random RNAi artifacts.

Further:

(3) Figure 4 H, I: Would Tau also aggregate in the absence of externally added Tau?

No. In the tau-Venus biosensor cell line, *SPHK2* knockdown alone did not increase tau-Venus foci formation (now Figure 3H, I). Tau-Venus foci increased only after addition of recombinant tau fibrils and were further enhanced by *SPHK2* knockdown. We now state this explicitly in the Results.

(4) How specific is the effect for Tau? It would help if the authors could assess other amyloid proteins.

We agree that similar membrane-level mechanisms may apply to other amyloid assemblies. We have therefore added recent literature to the Discussion supporting the broader concept that intralysosomal amyloid assemblies can physically deform and rupture lysosomal membranes. The revised manuscript states: “This interpretation is consistent with recent ultrastructural studies showing that intralysosomal amyloid assemblies can physically deform and rupture lysosomal membranes.” We further clarify that “whether this mechanism is specific to tau or also applies to other amyloid assemblies remains to be determined.”

Whether perturbation of SL metabolism similarly affects endolysosomal escape and seeded aggregation of other disease-associated amyloid proteins is an important question that we plan to address in future work. However, these experiments require additional disease-specific models, aggregation assays, and validation, and are therefore beyond the scope of the present revision.

(5) The connection between sphingolipids and AD is not new. See He et al, 2010, Neurobiol. Aging + numerous publications and also not between Tau seeding and lysosomal rupture: Rose et al., PNAS 2024 (that has been cited by the authors).

We agree and our manuscript does not aim to establish these associations as new. We state explicitly that alterations in sphingolipid metabolism have been reported in aging and AD, and that endolysosomal rupture is increasingly recognized as a critical step in tau seed escape and propagation.

The novelty of our study lies in mechanistically connecting these two previously established areas. Specifically, we show that genetic perturbation of enzymes involved in sphingolipid metabolism reduces endolysosomal membrane fluidity, promotes membrane rupture, and thereby increases susceptibility to tau seed escape and seeded aggregation. We have revised the Introduction and Discussion to better emphasize this mechanistic contribution.

**Recommendations for the authors:**

**Reviewer #1 (Recommendations for the authors):**

Figure formatting and annotation need improvement. Panel letters throughout the figures should be in uppercase, and gene names in pathway diagrams should be italicized for consistency. Several scale bars are missing, including in Figures 1C, 2A, and 2H, and should be clearly indicated in the figures and legends. In Figure 1C, the age of the worms used in the assay is not specified. While the Methods section mentions "age-synchronized animals," the precise age at the time of imaging or experimentation is not stated. It would strengthen the study to explore whether membrane integrity phenotypes vary between young adults (day 1) and older adults (day 7 or 10) across the different conditions. Figure 1B lacks sufficient detail describing the galectin puncta assay used. A brief explanation of the assay rationale and readout would help contextualize the findings.

We thank the reviewer for pointing this out. We have revised the figures and figure legends accordingly by standardizing panel labels, adding scale bars where missing, and providing the age of animals used in the assays. We also expanded the description of the galectin puncta assay in the Results to explain the rationale and readout of sfGFP::LGALS3 puncta formation.

Regarding the reviewer’s suggestion to compare young and aged animals, we agree that age-dependent changes in endolysosomal membrane integrity are an interesting question. However, the purpose of the present study was to investigate how perturbation of sphingolipid metabolism affects endolysosomal membrane fluidity and rupture under the assay conditions used in our original screen. A systematic comparison across aging is beyond the scope of the current revision. We have therefore clarified the animal ages used in the relevant figure legends and Methods.

In Figure S1A, the authors show co-knockdown of multiple genes, including one condition with simultaneous RNAi against four targets. Because different RNAi clones can vary in knockdown efficiency, it is important to provide validation of gene knockdown levels (e.g., by qRT-PCR) shown in both panels a and b.

We agree that RNAi efficiency can vary between clones and that this is particularly relevant for combinatorial RNAi experiments targeting two or more potentially redundant genes. We have added this limitation to the Results section and now state: “Because KD efficiency was not assessed for the individual RNAi clones or co-RNAi combinations, these experiments do not allow comparison of relative RNAi strength or inference of the relative importance of individual genes. Thus, the conclusions drawn from these RNAi experiments are qualitative: specific single or combined KDs can promote endolysosomal rupture, whereas the absence of a detectable phenotype after RNAi cannot exclude gene involvement, as KD may have been insufficient.”

Where mutant strains were available, we performed genetic validation. Specifically, a *sphk-1* mutant available at CGC (CZ24969; *sphk-1*(*ju831*)) also showed increased hypodermal sfGFP::LGALS3 puncta (new Figure S1A), supporting the RNAi-based conclusion that genetic perturbation of sphingolipid metabolism compromises endolysosomal integrity. Corresponding mutant strains were not available for the other selected hits. Importantly, most hits also induced sfGFP::LGALS3 foci in human HEK293T cells as assessed in our previous study [1], providing additional support that the observed effects are not random RNAi artifacts.

In Figure 2E, the FRAP recovery curves show only ~60% recovery in controls after 25 seconds, and an even lower recovery (~40%) in hpo-8 and spp-10 RNAi conditions. The authors should discuss why the recovery is incomplete and what it implies about the mobile fraction of the protein or membrane components in these conditions.

We agree that incomplete FRAP recovery is informative. For this reason, we report both the time to half-maximal recovery (thalf) and the maximal recoverable fluorescence signal. Increased thalf indicates reduced lateral mobility of LAAT-1::mCherry within the lysosomal membrane, consistent with reduced membrane fluidity. In addition, a reduced maximal recovery suggests that a larger fraction of the reporter is immobile or only slowly mobile during the time window analyzed. This may reflect stronger confinement of LAAT-1::mCherry within even more rigid membrane domains. However, because RNAi efficiency may differ between clones and we have not assessed their individual KD efficiency, we avoid overinterpreting differences in the absolute strength of recovery defects between individual KDs. Instead, we conclude that KD of sphingolipid-metabolism genes identified in our screen consistently reduces lysosomal membrane fluidity, as reflected by increased thalf and, in some cases, reduced maximal recovery.

In Figure S3A, the Western blot for SPHK2 shows unequal loading between the control and siSPHK2 lanes. The blot should be normalized to a loading control and quantified to demonstrate knockdown efficiency.

We have quantified SPHK2 levels relative to GAPDH across independent experiments and present the normalized quantification (Figure S3C-E).

Key experimental details are missing from the manuscript. The strains of *C. elegans* and RNAi bacteria used were not described, and there is no information on biological replicates. The authors should clarify how many times each experiment was performed and provide more transparency on experimental reproducibility.

We thank the reviewer for pointing this out. The *C. elegans* strains and RNAi bacterial clones used in this study were established and fully described in our previous study, which has now been published in Autophagy [1]. We now cite the published article throughout the revised manuscript and have added additional information in the Results section to explain the key features of the strains used here.

We have also revised the Methods and figure legends to improve transparency regarding experimental details. The figure legends include the number of biological replicates, the number of animals or cells analyzed, and the statistical tests used for each experiment. In addition, the Statistical Analysis section in the Methods now summarizes how replicate numbers and sample sizes are reported across the study. Finally, the source details for the strains and RNAi clones used in this study are now provided in Tables S1 and S2, respectively. These revisions should improve the experimental clarity and reproducibility of the data shown.

References:

(1) Sandhof CA, Martin N, Tittelmeier J, Schlueter A, Pezzali M, Schoendorf DC, et al. A novel *C. elegans* model for MAPT/Tau spreading reveals genes critical for endolysosomal integrity and seeded MAPT/Tau aggregation. Autophagy. 2025;21(12):2963-81. Epub 20250904. doi: 10.1080/15548627.2025.2551676. PubMed PMID: 40851193; PubMed Central PMCID: PMCPMC12758218.

(2) Yong J, Villalta JE, Vu N, Kukurugya MA, Olsson N, Lopez MP, et al. Impairment of lipid homeostasis causes lysosomal accumulation of endogenous protein aggregates through ESCRT disruption. eLife. 2024;12. Epub 20241223. doi: 10.7554/eLife.86194. PubMed PMID: 39713930; PubMed Central PMCID: PMCPMC11666243.

(3) Calixto A, Chelur D, Topalidou I, Chen X, Chalfie M. Enhanced neuronal RNAi in *C. elegans* using SID-1. Nat Methods. 2010;7(7):554-9. doi: 10.1038/nmeth.1463. PubMed PMID: 20512143; PubMed Central PMCID: PMC2894993.

(4) Li Y, Zhang J, Li M, Yang L, Wang X. Sphingosine kinase SPHK-1 maintains sphingolipid metabolism to protect lysosome membrane integrity in *C. elegans*. Mol Biol Cell. 2026;37(1):ar1. Epub 20251105. doi: 10.1091/mbc.E25-04-0182. PubMed PMID: 41191545; PubMed Central PMCID: PMCPMC12696880.
